# A fundamental study on the degradation of paracetamol under single- and dual-frequency ultrasound

**DOI:** 10.1016/j.ultsonch.2023.106320

**Published:** 2023-02-06

**Authors:** Mehrdad Zare, Pello Alfonso-Muniozguren, Madeleine J. Bussemaker, Patrick Sears, Efraím A. Serna-Galvis, Ricardo A. Torres-Palma, Judy Lee

**Affiliations:** a School of Chemistry and Chemical Engineering, University of Surrey, Guildford GU2 7XH, United Kingdom; b Grupo de Investigación en Remediación Ambiental y Biocatálisis (GIRAB), Instituto de Química, Facultad de Ciencias Exactas y Naturales, Universidad de Antioquia UdeA, Calle 70 # 52-21, Medellín, Colombia; c Grupo de Catalizadores y Adsorbentes (CATALAD), Instituto de Química, Facultad de Ciencias Exactas y Naturales, Universidad de Antioquia UdeA, Calle 70 # 52-21, Medellín, Colombia

**Keywords:** Pharmaceuticals, Sonodegradation, Cavitation, Dual-frequency ultrasound, Dosimetry, Sono(chemo)luminescence

## Abstract

The degradation of paracetamol, a widely found emerging pharmaceutical contaminant, was investigated under a wide range of single-frequency and dual-frequency ultrasonic irradiations. For single-frequency ultrasonic irradiation, plate transducers of 22, 98, 200, 300, 400, 500, 760, 850, 1000, and 2000 kHz were employed and for dual-frequency ultrasonic irradiation, the plate transducers were coupled with a 20 kHz ultrasonic horn in opposing configuration. The sonochemical activity was quantified using two dosimetry methods to measure the yield of HO• and H_2_O_2_ separately, as well as sonochemiluminescence measurement. Moreover, the severity of the bubble collapses as well as the spatial and size distribution of the cavitation bubbles were evaluated via sonoluminescence measurement. The paracetamol degradation rate was maximised at 850 kHz, in both single and dual frequency ultrasonic irradiation. A synergistic index higher than 1 was observed for all degrading frequencies (200–1000 kHz) under dual frequency ultrasound irradiation, showing the capability of dual frequency system for enhancing pollutant degradation. A comparison of the results of degradation, dosimetry, and sonoluminescence intensity measurement revealed the stronger dependency of the degradation on the yield of HO• for both single and dual frequency systems, which confirms degradation by HO• as the main removal mechanism. However, an enhanced degradation for frequencies higher than 500 kHz was observed despite a lower HO• yield, which could be attributed to the improved mass transfer of hydrophilic compounds at higher frequencies. The sonoluminescence intensity measurements showed that applying dual frequency ultrasonic irradiation for 200 and 400 kHz made the bubbles larger and less uniform in size, with a portion of which not contributing to the yield of reactive oxidant species, whereas for the rest of the frequencies, dual frequency ultrasound irradiation made the cavitation bubbles smaller and more uniform, resulting in a linear correlation between the overall SL intensity and the yield of ROS.

## 1 Introduction

In recent years, thanks to the advancements achieved in analytical chemistry, the detection and quantification of trace organic pollutants have been feasible up to the order of ng/L [1], [2], [3]. This advancement has revealed unregulated compounds being released to the environment [4] that current municipal water treatment plants are unable to entirely capture and degrade [5], [6], [7], [8] and consequently, allowing such compounds (at trace levels) to be discharged and built up in the water cycle and appear even in the drinking water [9]. These trace pollutants, called emerging contaminants (EC), include chemicals such as industrial additives, plasticizers, dyes, flame retardants, surfactants, perfluorinated compounds, and pharmaceuticals [1], [10] which are increasingly consumed to improve human life expectancy and quality [11], [12]. Recent studies have shown pharmaceutical ECs have a direct and indirect harmful impact on the environment and human health [1], [13]. Studies on algae, invertebrates, and fish have evidenced that these pollutants adversely affect endocrine and reproduction cycles, water transport, and osmoregulation processes of biota, changing the cellular reactions of vital organs or gene expressions of living creatures [14], [15], [16], [17], [18], [19]. Some pharmaceuticals are widely consumed antibiotics that could inhibit biodegradation processes and if discharged could lead to bacterial resistance in the environment and consequently, the spread of diseases [20], [21].

Considering the inadequacy of current water treatment plants for the removal and degradation of ECs, especially pharmaceuticals, alternative treatment methods have been explored to address this issue [22]. Studies have shown ultrasound irradiation is an effective and promising advanced oxidation process (AOP) for the degradation of organic pollutants and more specifically, pharmaceuticals [23].

During ultrasound irradiation of solutions, if the acoustic pressure is strong enough, existing gas nuclei within the solution will grow via rectified diffusion and coalescence under Bjerknes forces, until they reach the active size at which an adiabatic collapse occurs [24]. The collapse results in severe pressure and temperature (∼5000 K and ∼ 1000 bar) inside the bubbles [25] causing molecules of the water vapour or oxygen inside the bubbles dissociate to form HO•. The radical is well-known as a strong reactive oxidant species (ROS) that could directly attack and degrade the organic molecules within the solution [24]. The radicals could also combine and produce H_2_O_2_ which is a weaker ROS [26]. In addition to the chemical oxidation by the ROS produced, organic pollutants might be degraded via two other mechanisms (by pyrolysis inside/at the surface of the bubbles as well as supercritical water oxidation near the surface of the bubbles) [27]. During the bubble growth or collapse, organic pollutant molecules near the cavitation bubble surface could enter the bubble core via evaporation [28] or injection of nanodroplets into the bubbles [29]. In this condition, the organic pollutants would be directly pyrolyzed under the severe conditions of the adiabatic bubble collapse. Moreover, the extreme pressure and temperature generated inside a collapsing bubble could lead to the formation of a zone of supercritical water near the bubble surface, which could contribute to further degradation of the organic pollutants via thermolysis. However, in the vicinity of the bubbles, the oxidative effect of ROS is expected to be dominant and the thermolysis by the supercritical water would be negligible.

While ultrasound is known as a clean AOP that does not require the addition of any chemical, the main drawbacks of this method are high energy consumption and long reaction times [30]. Accordingly, studies have been aiming to address these issues by finding the optimum condition via investigation of different parameters such as the sonication frequency and power [31], [32], or combining ultrasound with other AOPs or other ultrasound frequencies to enhance the efficiency of the process by the possible synergy [33], [34], [35]. Considering that combining multiple ultrasound frequencies requires the addition of no chemicals, since the early 2000 s, there has been an increase in the investigation of the possible advantages that simultaneous application of multiple frequencies might have over single-frequency ultrasound (SFUS). Several studies have reported a synergistic effect for the application of dual-frequency ultrasound (DFUS) [36] and have attributed this synergy to the magnification of the bubbles’ nonlinear oscillation under the combined and simultaneous resonances of the input frequencies [37]. Accordingly, an index could be defined to quantify the synergistic effect (Equation 1) [38]:

(1) $$\mathrm{SI}=\;\frac{E_{\mathrm{DFUS}}}{E_{f_{1}}+\;E_{f_{2}}}$$

where *SI* is the synergistic index, *E* is any measurable effect such as ROS yield or pollutant degradation rate, and *f_1_* and *f_2_* are either of the input frequencies of the DFUS. Studies have investigated various conditions and parameters, reporting different *SI* [30], [39], [40], [41], [42], [43], [44], [45] ranging from above 1 [42], [46] to above 3 [40], and in some cases contradicting results [47], [48]. Several studies have shown that including one low-frequency ultrasound (<100) in multi-frequency irradiation is beneficial in terms of sonochemistry because of the low cavitation threshold at low frequencies [40], [41], [44], [45], [46], [49], [50], [51]. Also, Lee and Oh [41] have shown that the synergistic effect from dual frequency systems could be maximized if the transducers were located in opposing configurations when a high-frequency ultrasound is involved in DFUS with low to moderate powers.

To the best of the authors' knowledge, a majority of the studies have covered only a limited range of operating conditions and parameters such as frequency, and consequently, the relationship between the synergistic effect and sonication parameters is still unclear and requires further research. Most of the studies on multi-frequency sonication have covered fundamentals such as oxidant yield [45], sonoluminescence (SL) [46] or sonochemiluminescence (SCL) [44], and bubble size and dynamics [37]. There are limited reports on DFUS for the degradation of pollutants that focuses on the combination of frequencies that extends to frequencies below 100 kHz [48], [52], [53], which are known for mechanical rather than sonochemical effects [54]. Also, to the best of the authors' knowledge, there are currently no systematic studies that have investigated the correlation between the fundamental characteristics of dual-frequency sonication and the degradation of pollutants, especially pharmaceuticals.

In this study, the application of ultrasound for the degradation of paracetamol (PCM) was investigated. PCM is an over-the-counter (OTC) medicine with unregulated consumption, which has widely been found as an EC in water/wastewater [55]. In order to characterize and compare single- dual-frequency sono-reactors and assess their capabilities and any possible synergistic effect in the degradation of pharmaceutical emerging contaminants, a wide range of ultrasound frequencies were utilised as SFUS systems, also in conjunction with a 20 kHz ultrasound horn as DFUS systems, and the possible synergy in the generation of the ROS as well as degradation of PCM was studied. In addition, further experiments were conducted to quantify the sono- and sonochemi-luminescence intensity for the SFUS and DFUS ultrasonic systems to achieve deeper insight into their differences and the mechanisms involved in the degradation of paracetamol.

## 2 Materials and methods

### 2.1 Chemicals

Potassium iodide (KI, purity ≥ 99.5 %) was obtained from Fisher Chemicals. Ammonium molybdate tetrahydrate ((NH_4_)_6_Mo_7_O_24,_ purity ≥ 99 %), Sodium Hydroxide (NaOH, purity ≥ 98 %), and Luminol (5-Amino-2,3-dihydro-1,4-phthalazinedione, purity ≥ 97 %) were obtained from Sigma-Aldrich. Paracetamol (PCM, 4-Acetaminophenol, purity ≥ 98 %) was purchased from ACROS Organics and used as received. All solutions were prepared using Milli-Q water (18.2 MΩ). Methanol, acetonitrile, water, and formic acid Optima^TM^ grade (purity ≥ 99.9 %) from Fisher Chemicals, were used as the mobile phase for liquid chromatography/mass spectrometry (LCMS).

### 2.2 Experimental setup

**Fig. 1:**
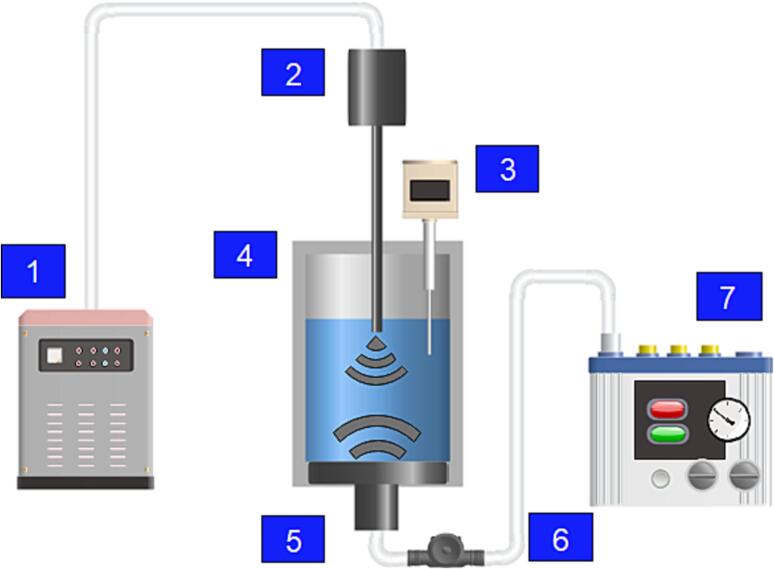
The schematic view of the experimental setup: 1) Power amplifier, 2) Ultrasonic horn, 3) Thermometer, 4) Sonication reactor, 5) Ultrasonic plate transducer, 6) Impedance matching unit, and 7) Power Amplifier.

The reactor used was a jacketed glass cylinder with an internal diameter of 6.7 cm and a plate transducer mounted at the bottom (Fig. 1). For SFUS irradiation, only the plate transducer of a given frequency (22, 98, 200, 300, 400, 500, 780, 850, 1000, and 2000 kHz, supplied by Honda Electronics transducers) was used, each comprising a 5 cm in diameter piezo-electric ceramic, connected to a 10 cm in diameter vibration plate. For DFUS, a 20 kHz ultrasonic horn (Fisherbrand™ Q700 Sonicator) was inserted into the reaction chamber from the top, in the opposite configuration with respect to the plate transducer. For each plate transducers from 200 to 2000 kHz, a constant amplifier load power of 20 W was applied, and due to the losses, the calorimetric power of 16.74 W was delivered to the solution (41.85 W/L). However, for 22 and 98 kHz transducers, the measured calorimetric power was 11.14 W (27.85 W/L), which was the result of the difference in the electrical structure, compared to the rest of the transducers. For DFUS, the ultrasonic horn was used with 30 % of its maximum amplitude which alone resulted in the calorimetric power of 33.42 W (83.55 W/L). i.e., under DFUS conditions, the solution was subjected to a total colorimetric power of 50.16 W (125.4 W/L) at each frequency from 200 to 2000 kHz, and 44.56 W (111.4 W/L) for 22 and 98 kHz. For all experiments, a chiller was used to set the initial temperature of the solution to 16 °C prior to sonication, and maintain it during the sonication within 5 °C above the initial temperature.

### 2.3 Experimental and analytical methods

#### 2.3.1 Degradation of paracetamol

For each SFUS and DFUS experiment, 400 mL of 5 mg/L paracetamol solution was sonicated for 60 min. the PCM degradation was monitored using a UV–Visible spectrophotometer (UV–vis.)(Evolution 201 by Thermo Scientific) at 243 nm [56]. 2 mL samples were analysed every 10 min and returned to the solution to maintain the power density exerted on the solution. To confirm the spectrophotometric results as well as to account for the probable interference of degradation intermediates generated during sonication, the samples were also analysed using a liquid chromatography-mass spectrometry (LCMS) system.

#### 2.3.2 LCMS method development

An LCMS comprising Waters Acquity^TM^ Ultra-Performance Liquid Chromatograph (UPLC) equipped with a Kinetex® PS-C18 100 Å column (100 mm × 2.1 mm, 2.6 µm), followed by a Waters Zspray^TM^ single quadrupole mass spectrometer SQD2 (MS) was employed in positive electrospray ionisation (ESI + ) mode. The chromatographic separation of paracetamol from the degradation intermediates was conducted with a gradient method (Table 1) for 10 µL samples, while the column temperature was maintained at 40 °C. Accordingly, a mixture of 0.1 % (v/v) formic acid in ultrapure water (A) and acetonitrile (B), with a flow rate of 250 µL/min was utilised as the mobile phase for the chromatography.

**Table 1:** The details of the gradient method utilised for the chromatographic separation of PCM from degradation intermediates, with a constant flow rate of 250 µL/min. The A and B are 0.1 % (v/v) solutions of formic acid in ultrapure water, and acetonitrile, respectively.

Time (min)	% A	% B
0.00	95.0	5.0
0.50	95.0	5.0
3.00	50.0	50.0
4.00	50.0	50.0
5.00	1.0	99.0
5.50	1.0	99.0
7.00	95.0	5.0
8.00	95.0	5.0

Following the chromatographic separation, PCM was detected as the protonated ion ([M + H]^+^) at *m*/*z* = 152 using the MS, run in the Selected Ion Monitoring (SIM) mode, with a retention time of 2.3 min. The MS settings applied for the analysis are mentioned in Table 2.

**Table 2:** The settings applied to Waters Zspray^TM^ single quadrupole mass spectrometer SQD2 (MS), run in ESI + and SIM mode, for the detection of PCM.

Parameter	Unit	Value
Source Voltages		
Capillary	kV	3.00
Cone	V	20
Source Temperature	°C	400
Source Gas	–	Nitrogen
Source Gas Flow rate		30.0
Desolvation	L/h	500
Cone	L/h	50
Interface Heater	–	On

#### 2.3.3 ROS production

To quantify the concentration of the main ROS produced during the sonication (HO• and H_2_O_2_), two sets of dosimetry experiments were performed. For HO•, 400 mL of 0.1 M KI solution was sonicated under the same conditions as for degradation experiments, and 2 mL samples were taken at different times for the analysis. The generation of HO• follows the zero-order reaction kinetics model, meaning that the rate is constant and independent of time. Thus, the KI solution was sonicated only for 10 min to generate enough data points to calculate the rate constant accurately. To maintain the power density exerted on the solution, the samples were returned to the reactor after the analysis.

During the sonication, the iodide (I^-^) in the solution reacts with the generated HO• according to the following mechanism (Reaction 1 to Reaction 4), yielding I_3_^-^ [49]:$\mathrm{HO}\cdot +I^{-}\to {\mathrm{OH}}^{-}+I\cdot$Reaction 1$I\cdot +I^{-}\to I_{2}^{-}$ Reaction 2${2\;I}_{2}^{-}\;\to \;I_{2}+\;2\;I^{-}$ Reaction 3$I_{2}+\;I^{-}\to {I_{3}}^{-}$ Reaction 4

The concentration of I_3_^-^, which is proportional to the concentration of HO• was determined by measuring the absorption at the wavelength of 350 nm using a UV–vis. and based on the Beer-Lambert Law with a molar absorptivity (ε) of 26,000 L⋅mol^−1^⋅cm^−1^ and path length of 1 cm [57].

The H_2_O_2_ generated during sonication does not react instantly with the iodide in the KI solution, and therefore could not be accounted for in the KI dosimetry method [57], [58], [59]. Hence, the second set of dosimetry experiments was conducted using 0.5 mM ammonium molybdate in the 0.1 M KI solution, to account for hydrogen peroxide as well. The ammonium molybdate within the KI solution acts as a catalyst for Reaction 5 [57]:$H_{2}O_{2}+2I^{-}\to 2OH^{-}+I_{2}$ Reaction 5

The produced I_2_ via Reaction 4 then undergoes Reaction 5. Consequently, using this method the concentration of total ROS (both the HO• and H_2_O_2_) could be measured simultaneously. By subtracting the HO• yield measured using pure KI solution, the yield of H_2_O_2_ could be calculated.

To evaluate the accuracy of the H_2_O_2_ yield calculated using this method, the concentration of the H_2_O_2_ was measured for sonicated Milli-Q water based on method described by Alegria et al. [58] as well, under DFUS at 200, 500, and 850 kHz (Fig-S. 4 of the supplementary data).

#### 2.3.4 Sono- / sonochemi-luminescence intensity

To achieve a better insight into SFUS and DFUS acoustic fields, the spatial distribution and intensities of the SL and SCL generated during the sonication were recorded for each frequency, using an ANDOR iXon3 EMCCD camera and the software. For this purpose, 400 mL of Milli-Q water was photographed under sonication with the same condition as for the degradation experiments. For all SL images, the camera was operated at −70 °C, and the exposure time of 20 s as well as the Electron Multiplying (EM) Gain level of 100 were applied. The photography was conducted in a light-proof box to eliminate the noise of the ambient light. However, to omit the probable noise of any light diffusing into the photography box, background photographs were recorded before and after the sonication. Then the average light intensity recorded in the two background photographs was subtracted from the overall intensity of the main photograph.

To further assess the effect of the DFUS on the acoustic field, SCL measurement was conducted under SFUS and DFUS at three representative frequencies of 200, 500, and 850 kHz from the low-, intermediate- and high-frequency zones. For this purpose, 400 mL of 0.1 M NaOH solution containing 1 mM luminol was sonicated under the same condition as that of PCM degradation. The SCL photographs were taken via the same procedure mentioned above for the SL measurement, but due to the considerably higher brightness generated by the luminol, the exposure time of 4 *sec* and the EM Gain level of 4 were applied to the camera settings.

The light intensity recorded by the camera was also analysed pixel by pixel, by calculating the normal distribution function (NDF) for the count of occurrence of the SL intensities recorded by each camera pixel. For this analysis, pixels with intensities less than 400 a.u. were considered noise and were removed.

## 3 Results

### 3.1 Degradation of paracetamol

For PCM degradation, both pseudo-1st- and 2nd-order reaction kinetics models were applied (Fig-S. 2 and Table-S. 1 of the supplementary data) and a comparison of the statistical parameters of both models. The order of the kinetic model implies the degree of sensitivity of the reaction to the concentration of the reactants and the higher the order, the higher the sensitivity. Accordingly, the better conformity of the 2nd-order kinetic model suggests the high sensitivity of the sonodegradation of PCM to its bulk concentration. Sonodegradation is a heterogeneous reaction in which the reaction site is at the surface or inside of cavitation bubbles [60]. Hence, the transport of the pollutant molecules from the solution bulk towards the cavitation bubbles plays a vital role in the reaction rate, and the mass transfer resistance with respect to the pollutant characteristics such as the degree of hydrophilicity, might play a dominant role and be rate controlling.

**Fig. 2:**
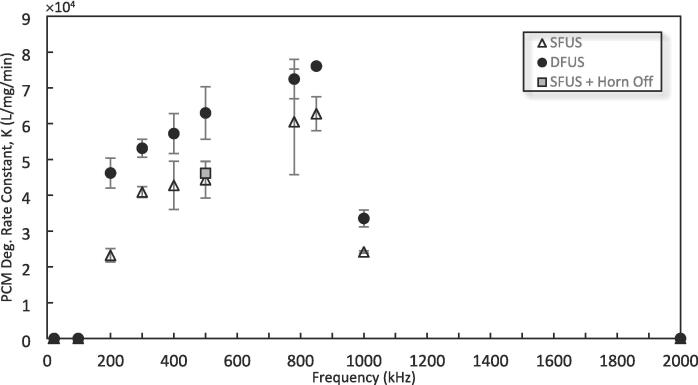
Pseudo 2nd order reaction rate constant for degradation of PCM under the studied condition as a function of the plate transducer frequency. The linear line is added to guide the eye.

The corresponding pseudo-2nd-order rate constants under SFUS and DFUS are presented in Fig. 2 versus the plate transducer frequencies. A comparison of the rates shows that the rate constant obtained for DFUS is higher than that for SFUS. For both SFUS and DFUS, the rate constant increases linearly with increasing frequency from 200 kHz to 850 kHz, especially for DFUS, before the drastic decrease at 1 MHz.

To make sure that the enhancement observed under DFUS is due to the 20 kHz ultrasound rather than the ultrasonic horn acting as a reflector, the SFUS at 500 kHz was repeated having the horn off within the solution. A comparison of the results (Fig. 2) shows that the effect of the horn as a reflector is negligible.

**Fig. 3:**
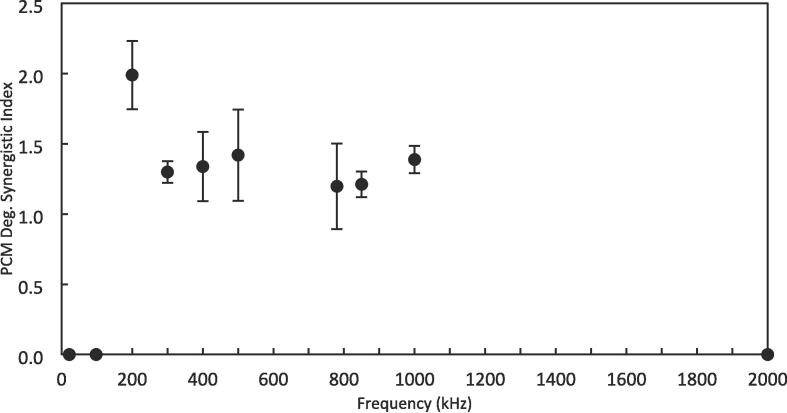
The synergistic index (SI) of the degradation of paracetamol by DFUS, calculated using Equation 1 and UV–vis. data, and plotted as a function of the plate transducer frequency.

The degradation synergistic index (SI) calculated based on Equation 1 is shown in Fig. 3. For all degrading frequencies, the SI is above one, meaning that introducing the 20 kHz horn to the acoustic field of the plate transducers leads to a synergistic effect for the degradation reaction, though no clear trend could be found in the graph.

PCM degradation under both SFUS and DFUS at 500 kHz was analysed using LCMS, and the degradation rate obtained accordingly was compared to that of the UV–vis. (Table 3). The comparison shows that for both SFUS and DFUS, the LCMS recorded an order of magnitude higher degradation rates. LCMS could specifically quantify PCM molecules whereas the UV–vis. measures the absorbance at the wavelength of 243 nm, which includes the absorbance by the intermediate molecules as well. Accordingly, it could be concluded that the data of the UV–vis. is beneficial in terms of simultaneous monitoring of PCM elimination as well as all the degradation intermediate sharing absorbance at 243 nm. Table 3 also shows that the difference between the rate constants under SFUS and DFUS is larger for the UV–vis. results. This confirms the higher degradation of intermediates under DFUS in comparison to SFUS. However, in terms of PCM molecules, LCMS measurements revealed no significant differences between SFUS and DFUS. It is worth mentioning that the pseudo-2nd-order reaction kinetics model showed better conformity with the LCMS results as well.

**Table 3:** The comparison of the pseudo 2nd order reaction rate constants calculated based on the results of UV–vis. and LCMS for SFUS and DFUS at 500 kHz. The details of the rate constant calculation for the LCMS results are presented in sections 2 and 3 of the supplementary data.

	Degradation Rate Constant, k (mg^−1^.L.min^−1^)	
	UV–vis.	LCMS
SFUS	4.44 × 10^-4^ ± 5.17 × 10^-5^	2.12 × 10^-3^ ± 5.52 × 10^-5^
DFUS	6.30 × 10^-4^ ± 7.34 × 10^-4^	2.43 × 10^-3^ ± 5.00 × 10^-6^

### 3.2 ROS dosimetry

**Fig. 4:**
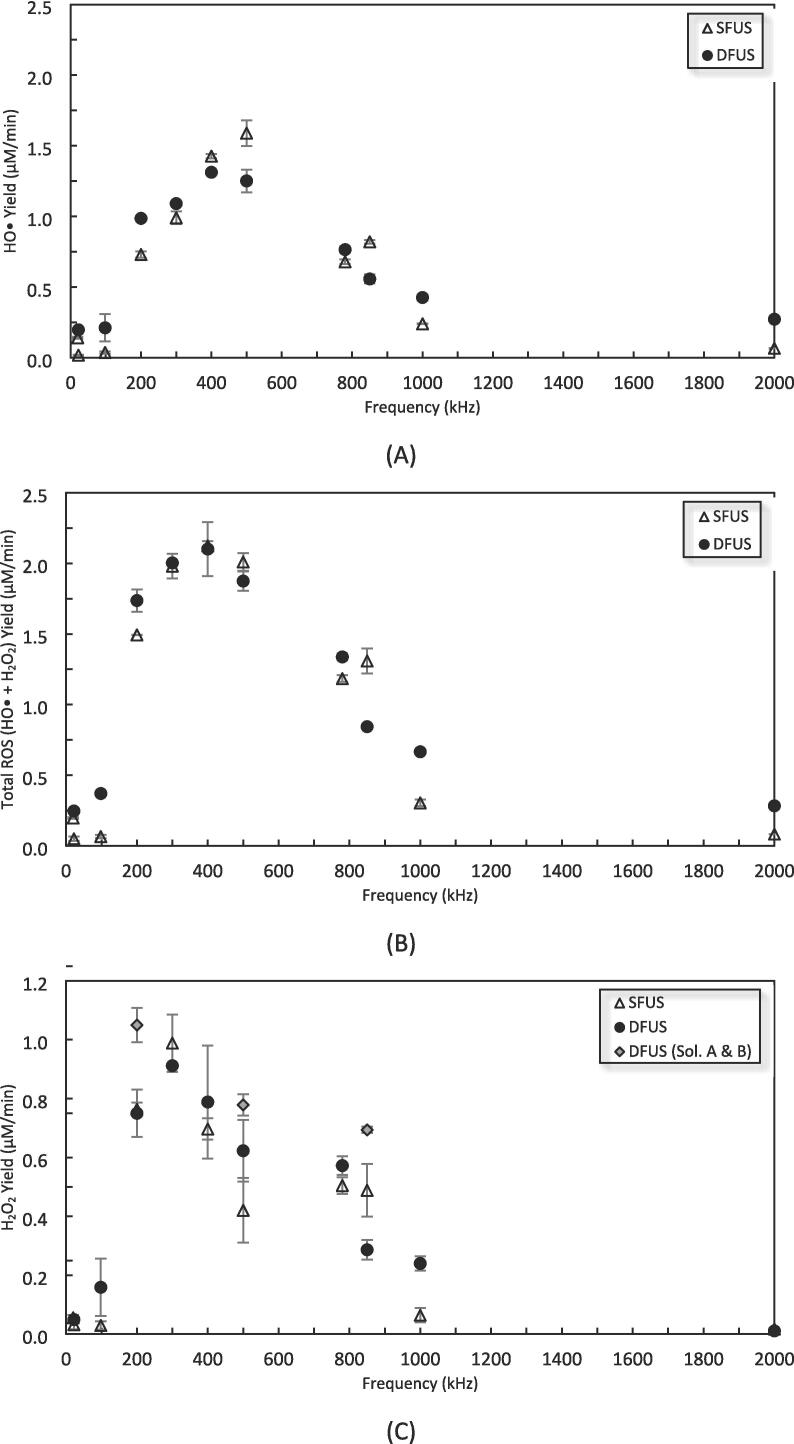
The dosimetry results for quantification of A) HO•, B) Total ROS, and C) H_2_O_2_, under SFUS and DFUS as a function of the plate transducer frequency.

Fig. 4 shows the yield of HO• and H_2_O_2_ for SFUS and DFUS at each frequency. The yield of HO• measured by KI dosimetry is shown in Fig. 4 (A), whereas Fig. 4 (B) shows the results of KI dosimetry with ammonium molybdate, which measures the concentration of both HO• and H_2_O_2_ simultaneously. By subtracting the two, the yield of H_2_O_2_ was achieved which is shown in Fig. 4 (C). The yield of H_2_O_2_ for both SFUS and DFUS, especially for the range of 200 kHz to 1 MHz in which degradation of PCM was observed, shows that a significant portion of HO• produced during sonication combined to form hydrogen peroxide. For 98 kHz, 200 kHz, 1 MHz and 2 MHz, applying DFUS enhanced the yield of HO• and total ROS. Also, Fig. 4 (B) reveals that for 300 and 400 applying DFUS did not make a considerable change to the yield of total ROS, whilst it slightly changed the HO• yield (Fig. 4 A), suggesting that DFUS could affect the HO• recombination and vary the yield of H_2_O_2_. It could also be seen that DFUS at 500 and 850 kHz reduced the yield of HO• as well as total ROS, suggesting a cancellation effect for the frequencies which is in agreement with previous studies [36]. The synergistic index for dosimetry results (either HO•, H_2_O_2_, or total ROS) versus frequency showed no specific trend (Fig- S. 2 in the supplementary data).

To validate the method used to obtain the yield of H_2_O_2_ shown in Fig. 4 (C), the method described by Alegria et al. [58], which is widely used as an ex-situ measurement method, was used to evaluate the H_2_O_2_ yield. A comparison of the results (Table-S. 3 of the supplementary data) showed that the results of both methods are acceptably close, showing the same trend.

### 3.3 Sono- and Sonochemi-luminescence intensity measurement

**Fig. 5:**
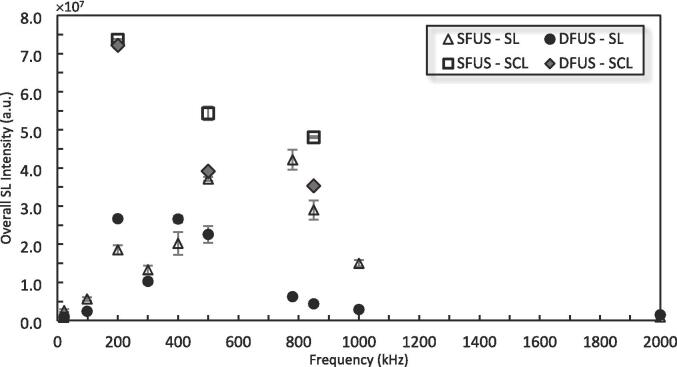
The overall sonoluminescence (SL) and sonochemiluminescence (SCL) intensity measured for SFUS and DFUS at different frequencies, as a function of the plate transducer frequency.

To quantify the cavitation collapse intensities as well as the sonochemical activity, the overall SL and SCL intensities were measured for SFUS and DFUS at each studied frequency (Fig. 5). Except for 200 and 400 kHz of the range of degrading frequencies, SFUS recorded a higher overall SL intensity than DFUS. Also, applying DFUS for degrading frequencies of 500 kHz and higher resulted in a drastic decrease in the overall SL intensity (by at least 80 %).

For SCL, applying DFUS did not make a considerable change at 200 kHz but at 500 and 850 kHz, it caused a drastic decrease in the SCL, the same as for the SL at these frequencies.

To evaluate the effect of the ultrasonic horn acting as a wave reflector, the SL and SCL intensity measurement experiments were repeated having the horn off within the reaction chamber. Similar results to those of SFUS were recorded in terms of overall SL and SCL intensities and spatial distribution (Fig-S. 6 and Fig-S. 7). Accordingly, it could be concluded that the effect of the ultrasonic horn is due to the introduction of the 20 kHz ultrasound to the acoustic field rather than the horn acting as a reflector.

**Fig. 6:**
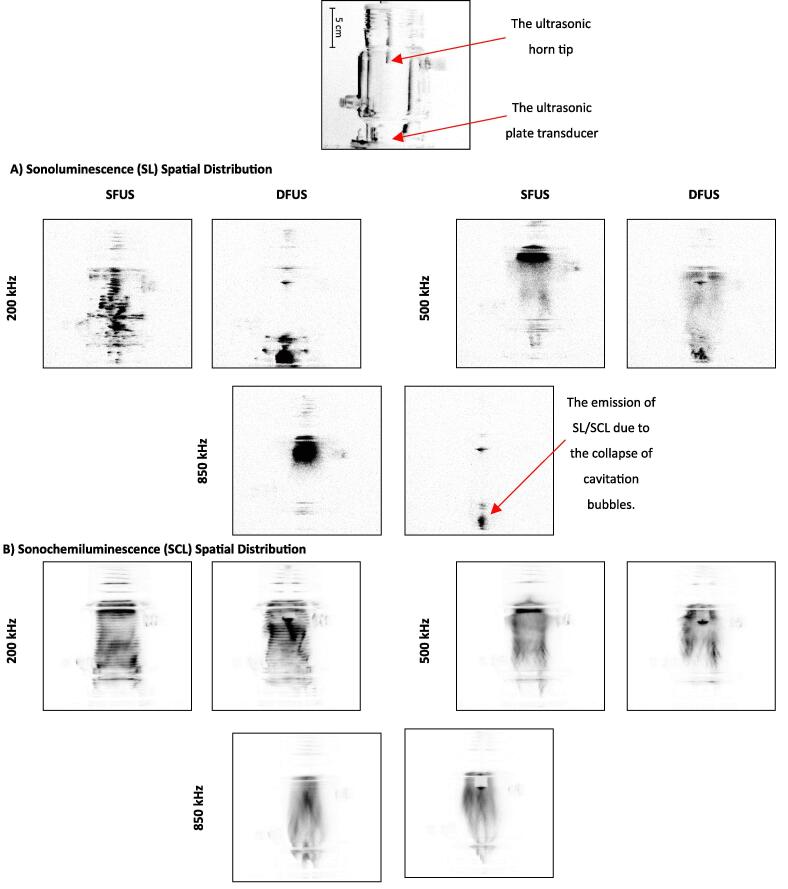
Spatial distribution of A) Sonoluminescence (SL), and B) Sonochemiluminescence (SCL) emission imaged for SFUS and DFUS at three distinguished frequencies. The same brightness/contrast settings are applied to the images to For better visibility the images are inverted, and the black spots represent the SL/SCL. The picture of the empty reactor is presented as a guide/scale.

Fig. 6 shows the qualitative comparison of SL and SCL intensity and spatial distribution for SFUS and DFUS at three distinguished frequencies (the results for the rest of the studied frequencies are presented in Fig-S. 8 of the supplementary data). Applying DFUS considerably changes the spatial distribution of SL within the acoustic field while having no significant effect on the SCL spatial distribution, showing that two distinct groups of cavitation bubbles contribute to SL and SCL [61]. DFUS generally pushes the SL-active region away from the solution surface and down towards the plate transducer. Specifically, for some of the frequencies such as 500 kHz and higher, DFUS enhances the SL spatial distribution by dispersing the cavitation bubbles throughout the reactor, though it reduces the overall SL intensity (Fig. 5). Along with the agitation by the ultrasound, the better dispersion of the cavitation bubbles throughout the reaction chamber could potentially further reduce the mass transfer resistance against the transport of the pollutant molecules towards the cavitation bubbles and enhance the degradation rate. Also, a comparison between the SL and SCL images reveals that the zone in which the majority of the SL bubbles lies is different from that for the SCL bubbles. Knowing that the SL is attributed to symmetric bubble collapses [62], [63] while SCL is emitted more from asymmetric collapses [61], [64], together with the effect of DFUS on the spatial distribution of SL and SCL bubbles, it could be concluded that DFUS more affects the formation and spatial distribution of stable cavitation bubbles collapsing symmetrically, rather than transient bubbles undergoing asymmetric collapse.

**Fig. 7:**
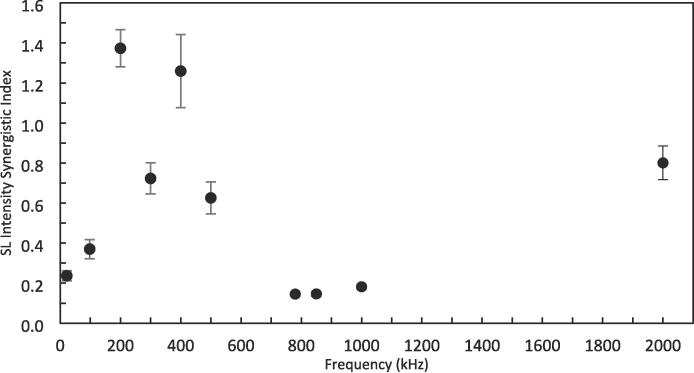
The synergistic index (SI) for the overall sonoluminescence (SL) intensities, calculated using Equation 1 and plotted as a function of the plate transducer frequency.

The synergistic index (SI) for the overall SL intensities at each frequency is presented in Fig. 7. For all frequencies but 200 and 400 kHz, DFUS results in a synergistic index < 1, which is due to the decrease in the overall SL intensity at the frequencies under DFUS (Fig. 5).

## 4 Discussion

There is a consensus that under SFUS there exists an optimum frequency at which a maximum degradation rate, ROS yield, SL and SCL intensities are observed [65], [66], [67], [68]. This is attributed to the balance between the increasing population of cavitation bubbles and decreasing bubble size and consequently the collapse intensity, as the sonication frequency increases [24]. However, the maximum degradation rate observed for PCM at 850 kHz under both SFUS and DFUS does not agree with the maximum yield of either of the ROSs, as well as the overall SL and SCL intensities. Moreover, PCM degradation under DFUS was significantly higher compared to SFUS resulting in a synergistic effect, whilst such an enhancement under DFUS was not observed for the ROS yields or the SL and SCL intensities. The enhancement observed in the degradation of PCM under DFUS could thus be attributed to the increase in mass transport of PCM molecules towards the cavitation bubble due to the introduction of the 20 kHz horn. This juxtaposition of mechanical and chemical (mechanochemistry) [69] effect may have led to the observed synergy.

To better understand the mechanisms involved in the sonodegradation of pharmaceuticals and the enhancement observed under DFUS, the correlations between HO•, H_2_O_2_, and SL/SCL are examined before assessing their impact on the degradation of PCM.

### 4.1 Correlation between HO•, H_2_O_2_, and SL intensity

**Fig. 8:**
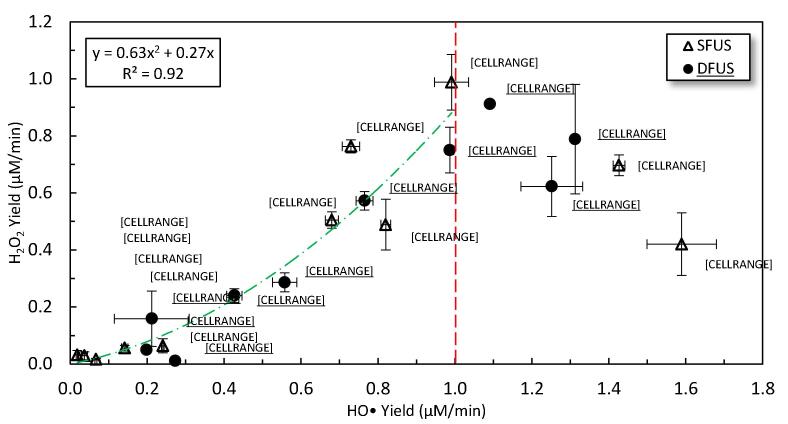
The correlation between total ROS and HO• generated during sonication, for both SFUS and DFUS. The red dashed line shows the threshold of 1 μM/min for the yield of HO• above which a decreasing trend forms and the green dotted dashed line shows the parabolic fit to the data below the threshold. (For interpretation of the references to colour in this figure legend, the reader is referred to the web version of this article.)

The correlation between HO• and H_2_O_2_ yield was examined by plotting the yield of H_2_O_2_ versus HO• yield (Fig. 8). Since the yield of H_2_O_2_ is 2nd order with respect to the concentration of HO• (d[H_2_O_2_]/dt = k [HO•][HO•]), a strong parabolic correlation (the green dotted dashed line) could be found below the threshold of 1 μM/min for the yield of HO•. Beyond this threshold (the red dashed line), the yield of H_2_O_2_ decreases despite the increases in the yield of HO•, suggesting consumption of H_2_O_2_.

At a high HO• yield (above the threshold of 1 μM/min), the HO• and the corresponding H• could start to attack the H_2_O_2_ via Reaction 6 and Reaction 7 [27]. Accordingly, the HO• is consumed and produced simultaneously while the H_2_O_2_ is permanently consumed to produce HO_2_•. It should be noted that HO_2_• (E°: 0.8–1.5 V) has lower oxidative potentials compared to HO• (E°: 1.8–2.7 V) or H_2_O_2_ (E°: 0.5–1.8 V), and hydroperoxyl radical would not significantly contribute to the degradation of organic pollutants [70], [71], [72], [73].

Reaction 6 $$H_{2}O_{2}+HO\cdot \to H_{2}O+HO_{2}\cdot$$

Reaction 7 $$H_{2}O_{2}+H\cdot \;\to H_{2}O+HO\cdot$$

**Fig. 9:**
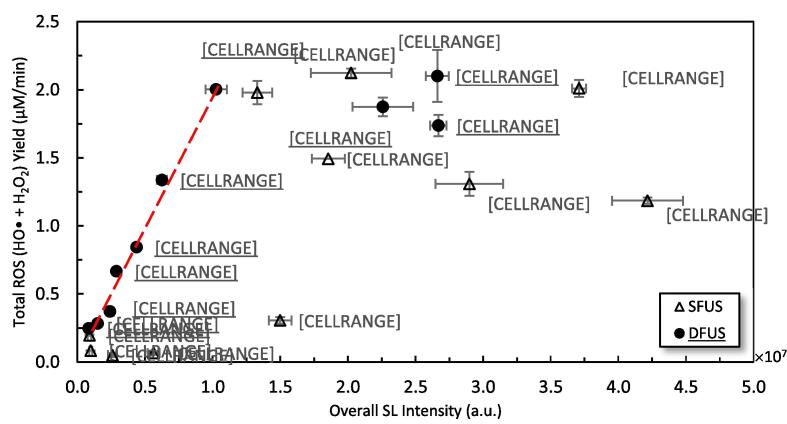
The relationship between SL and total ROS yield for SFUS and DFUS (the dashed line is added to assist the eye).

ROS yield is often considered to be proportional to SL intensity [74]. In contrast, the results in Fig. 4 and Fig. 5 do not appear to show such a correlation, which is also in agreement with another study [75]. DFUS appears to have a much more drastic impact on the SL emission than the ROS yield. For most frequencies, DFUS decreased the overall SL intensity whilst making no significant change in the yield of ROS. Fig. 9 shows the yield of total ROS versus the overall SL intensity. While no clear correlation could be found for SFUS, there is a strong linear correlation for DFUS for the overall SL intensities lower than 1 × 10^7^ a.u.. Above this threshold there appears to be a plateau where further increase in the SL does not increase the yield of ROS.

The SL emitted from a cavitation bubble depends on the bubble collapse temperature [24]. According to Equation (2), the bubble collapse temperature (T_Core_) is a function of the maximum (R_max_) and minimum (R_min_) bubble radius in an oscillation cycle, the bulk solution temperature (T_o_) and the specific heat ratio of the dissolved gases (γ) [24].

(2) $$T_{\mathrm{Core}}=T_{0}{\left(\frac{R_{\max}}{R_{\min}}\right)}^{3(\gamma -1)}$$

Therefore, for a constant solution temperature and dissolved gas concentration, T_Core_ and subsequently the emitted SL is proportional to the size of the collapsing bubble. i.e., for a constant solution condition, the intensity of the SL emitted by each bubble represents the bubble size and could be statistically analysed instead.

Accordingly, the individual bubble SL intensities, recorded by each camera pixel, were statistically analysed using the normal distribution function (NDF). Due to the proportionality described above, it is expected that the NDF for the individual SL intensities shows a similar trend and behaviour to that of the corresponding bubble sizes. In other words, a change in the extremum and the curve width of the NDF of the individual SL intensities would correspond to a similar change in that of the bubble sizes. It should be noted that the extremum and curve width of the NDF represent the arithmetic mean and the standard deviation of the analysed values respectively, which denote the average value and the degree of uniformity of the studied data, in this case, the individual SL intensities and the corresponding bubble sizes.

Active cavitation bubbles form at antinodes of the ultrasound frequencies and therefore, the population of the bubbles depends on the number of antinodes. For frequencies at which significant ROS was measured (200 kHz-1 MHz), introducing the 20 kHz ultrasound does not affect the number of the antinodes significantly as the number of the antinodes of these frequencies is multiple times that of a 20 kHz ultrasound i.e., the lowest degrading frequency (200 kHz) has 10 times more antinodes than 20 kHz and for higher frequencies, this ratio is even higher (∼43 times for 850 kHz). Therefore, it could be assumed that DFUS does not change the populations of cavitation bubbles significantly, compared to SFUS. In addition, the similarity in the ROS yield under SFUS and DFUS as well (Fig. 4) further supports the assumption that the active bubble population remains the same under both SFUS and DFUS. This allows for the comparison of SFUS with DFUS in terms of NDF of the individual SL intensities.

**Fig. 10:**
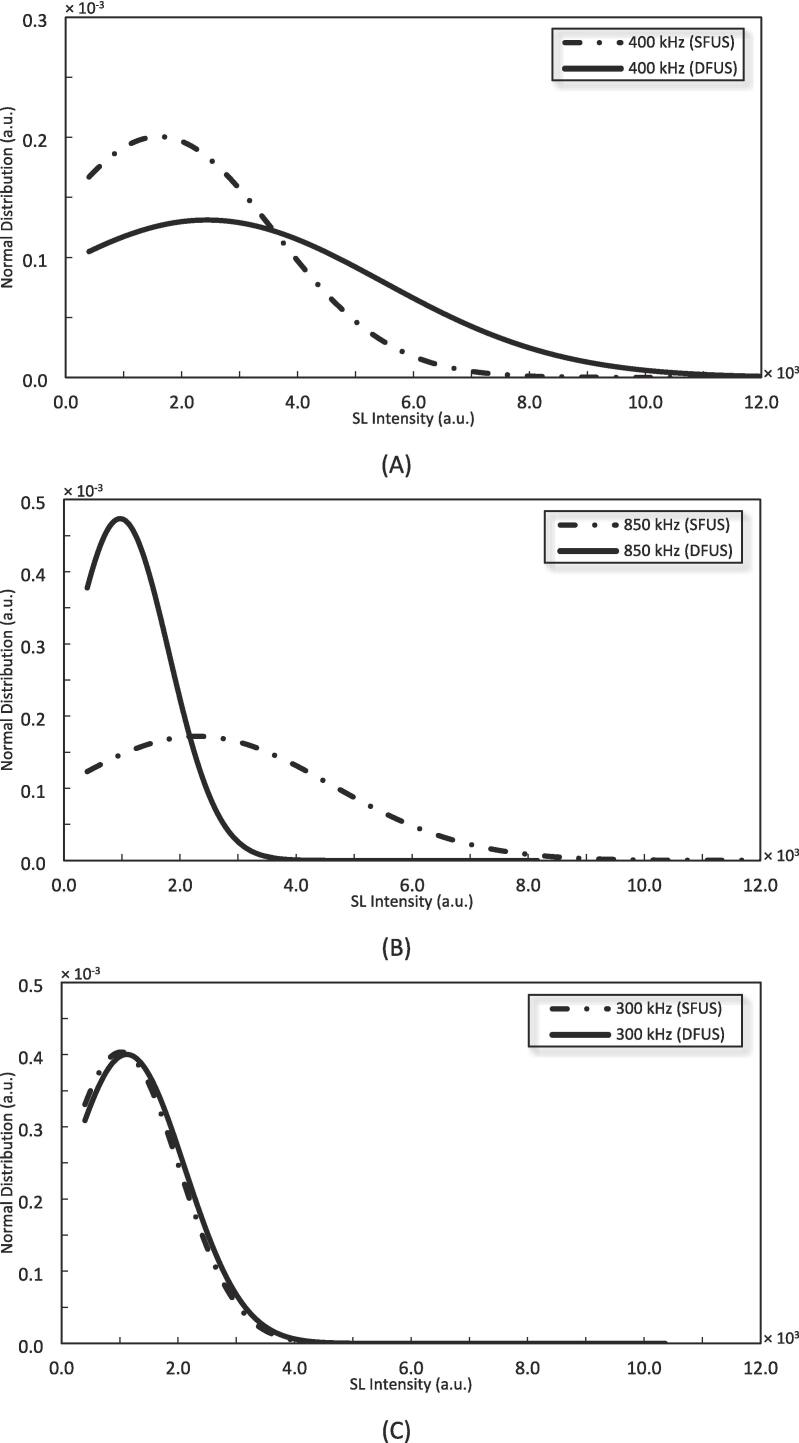
The normal distribution function (NDF) for the individual sonoluminescence (SL) intensities measured by each camera pixel under SFUS and DFUS at A) 400 kHz, B) 850 kHz, and C) 300 kHz.

Fig. 10 compares the NDF curves for three representative frequencies of 400 kHz, 850 kHz, and 300 kHz under SFUS and DFUS, respectively from the plateau, the linear section and the intersection of the two zones in Fig. 9 (the curves for the rest of the frequencies are presented in Fig-S. 9 and Fig-S. 10 of the supplementary data).

Accordingly, Fig. 9 and Fig. 10 (A) show that for 400 kHz, DFUS not only increases the overall SL intensity but also increases the average bubble radius as the extremum of the DFUS NDF curve is pushed to the right, compared to SFUS. Also, DFUS makes the curve wider, suggesting a wider range of diameters for the corresponding cavitation bubbles. The same effect was observed by DFUS for 200 kHz (Fig-S. 9 of the supplementary data), at which the overall SL intensity was increased under DFUS (Fig. 9). This suggests that DFUS at the frequencies promotes the growth of the bubbles, resulting in larger and more diverse bubble diameters.

According to Brotchie et al. [76], there is a range of bubble sizes in which bubbles emit SL but are too large to generate ROS. Accordingly, the majority of the large bubbles formed at 200 and 400 kHz under DFUS do not considerably contribute to the generation of ROS, despite increasing the overall SL intensity. Hence, the plateau forms in Fig. 9, as the increase in the overall SL intensity does not lead to a change in the yield of total ROS.

For 850 kHz and other frequencies of the linear section of the DFUS curve in Fig. 9 (where total ROS yield increases with overall SL intensity), an opposite effect was observed by DFUS. DFUS at these frequencies reduced the overall SL intensity (Fig. 9) and shifted the extremum of the NDF curve to the left (Fig. 10 B) and reduced the width of the curve as well, suggesting a decrease in the average size and size diversity of the cavitation bubbles. In conclusion, for these frequencies DFUS restricts the growth of cavitation bubbles, resulting in smaller and more uniform bubbles. It is also supported by the SCL data, as DFUS decreases the SCL overall intensity at 850 kHz. Considering that the asymmetric collapse of large bubbles plays a major role in SCL emission [61], [64], the decrease in the SCL intensity by DFUS could be due to the reduction in the size of the bubbles, which makes the bubbles more stable [77]. Due to the decrease in size, the majority of the SL emitting bubbles fall within the size range that also contributes to the production of ROS, resulting in the observed linear correlation between the overall SL intensity and the yield of ROS.

It is noteworthy that 500 kHz shows a similar behaviour under DFUS, despite lying in the plateau of Fig. 9. Unlike the other two frequencies in the plateau (200 and 400 kHz), DFUS at 500 kHz reduces the overall SL intensity (Fig. 9) as well as makes the cavitation bubbles smaller and more uniform. As a result, DFUS makes the data point of 500 kHz closer to the linear section, like for the frequencies of the linear section of Fig. 9. The NDF curves for 500 kHz under SFUS and DFUS are presented in Fig-S. 10 of the supplementary data.

For 300 kHz at the intersection of the linear and the plateau sections (Fig. 9), DFUS makes no considerable change in the overall SL intensity (Fig. 9), average bubble size and size distribution (Fig. 10 C).

It is worth mentioning that the NFD analysis for the SCL data did not show such a distinct meaningful behaviour (Fig-S. 11 of the supplementary data).

### 4.2 Correlation between degradation of paracetamol with HO• and SL

When the degradation of PCM is plotted against HO• and SL intensity with all the data together (Fig-S. 12), a strong correlation was not observed. However, considering the respective thresholds of HO• and SL at 1 μM/min (Fig. 8) and 1 × 10^7^ a.u. (Fig. 9), the degradation of PCM could be investigated under two different zones of “Low ROS / SL” (below the thresholds) and “High ROS / SL” (above the thresholds), which gives a clearer view to the data.

#### 4.2.1 Low ROS and SL zone

**Fig. 11:**
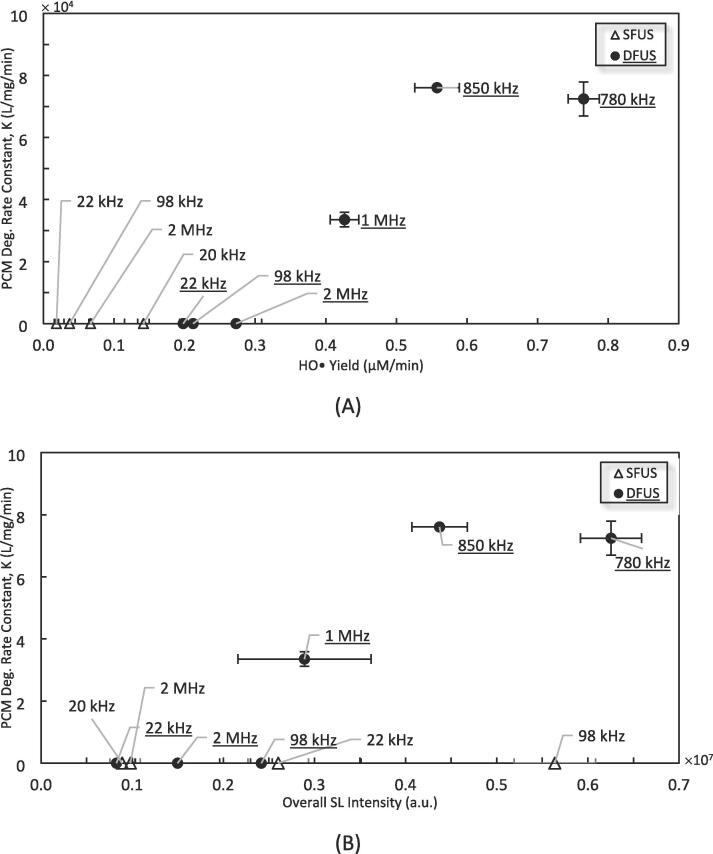
The relationship of PCM degradation with A) HO• Yield, and B) the overall SL intensity, for the Low ROS or SL zone (ROS lower than 1 μM/min or SL lower than 1 × 10^7^ a.u.).

The PCM degradation is plotted against the yield of HO• and overall SL intensity are shown in Fig. 11 (A) and (B), respectively. In both figures, a threshold is observed in HO• yield and SL intensity above which a significant degradation of paracetamol occurs (approximate HO• yield of 0.3 μM/min and overall SL intensity of 0.2 × 10^7^ a.u.). However, for SFUS 98 kHz, despite the overall SL intensity being above the threshold, no PCM degradation is recorded. The high SL intensity for this frequency could be attributed to the severe collapse of large bubbles that according to Brotchie et al. [76] might not necessarily contribute to the generation of ROS. Thus, the number of active cavitation bubbles and subsequently, the yield of ROS generated at this frequency is low, in comparison to higher frequencies.

The similarity of the trends observed in Fig. 11 (A) and (B) for PCM degradation (α) could be attributed to the linear relationships of HO• (*β*), total ROS (γ), and overall SL intensity (δ) in the low ROS and SL zones depicted in Fig. 8 and Fig. 9 (α∝βandβ∝γandγ∝δ,thereforeα∝δ). This suggests that in the low ROS and SL zone, the degradation of PCM is more likely to occur via chemical oxidation by the ROS rather than pyrolysis inside the bubble, which is in agreement with the low volatility of PCM (6.29 × 10^-5^ mm Hg at 25 °C) [78]. The oxidative mechanism is confirmed by Isariebel et al., by inhibiting the PCM degradation via adding *n*-butanol to the solution, as a HO• scavenger [79]. Also, this finding is in agreement with other studies reporting chemical oxidation by HO• as the main degradation mechanism, for more hydrophobic or even volatile organic compounds such as Levofloxacin and phenol [80], [81], [82], [83].

#### 4.2.2 High ROS and SL zone

**Fig. 12:**
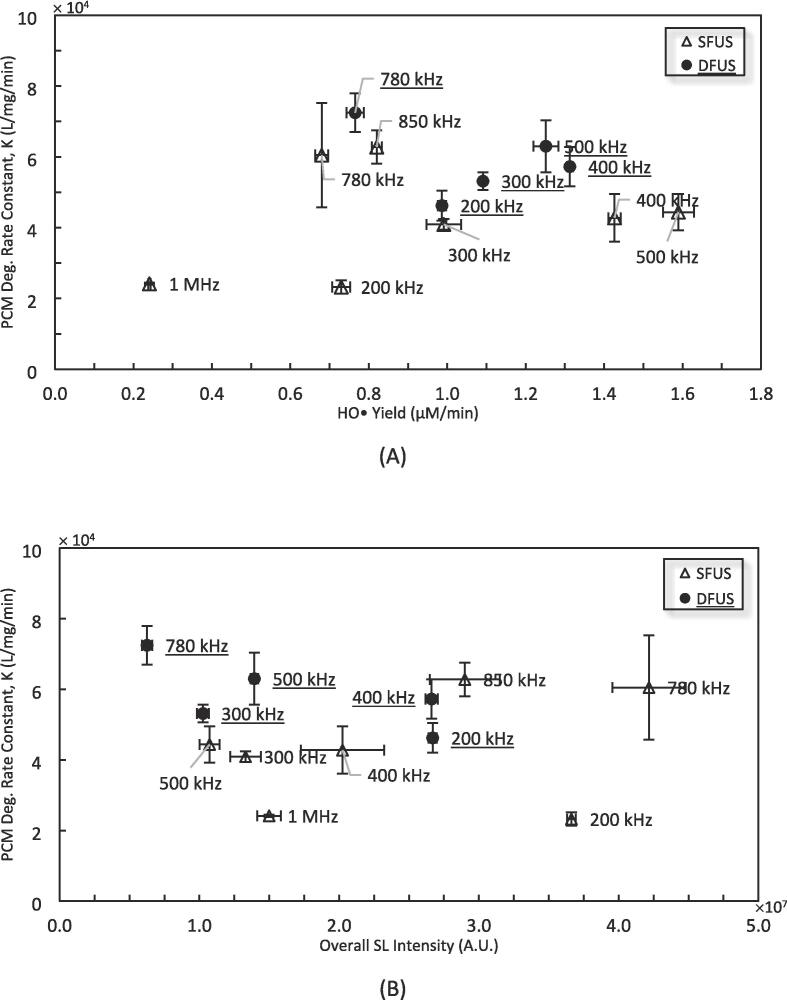
The PCM degradation versus A) HO• Yield, and B) the overall SL intensity, for the High ROS / SL zone (ROS above 1 μM/min or SL above 1 × 10^7^ a.u.).

The PCM degradation is plotted versus the HO• yield and the overall SL intensity in Fig. 12 (A) and (B) respectively. According to Fig. 12 (A), two distinct trends could be seen for frequencies lower and higher than 500 kHz. For the frequencies ≤ 500 kHz, the PCM degradation rate constant increases linearly with the yield of HO• and then decreases above the threshold of 1.14 μM/min for HO•. The linear increase reveals that the main mechanism for the degradation of PCM involves HO•, which is in agreement with the literature [79]. The linear decrease could be explained based on the decreasing trend above a certain threshold of HO• yield in Fig. 8, where the H_2_O_2_ scavenges the HO•. The low availability of the pollutant molecules in the vicinity of cavitation bubbles results in H_2_O_2_ winning the competition for reacting with HO•. This is in agreement with the results of Im et al. where the addition of H_2_O_2_ to PCM solution resulted in a decrease in the degradation rate, beyond a certain threshold [84].

For frequencies > 500 kHz, a similar linear increase could be observed, giving similar degradation rates for lower HO• yields. This observed enhancement could be explained by the enhanced mass transfer under higher frequencies. It is well understood that sonodegradation is a heterogeneous reaction in which the reaction site is the surface or inside of cavitation bubbles [60]. Hence, it is highly sensitive to the transport of the pollutant molecules towards the bubbles, and depending on the mass transfer resistance, the degradation reaction could be diffusion-controlled. Therefore, enhancing the mass transfer could result in an increase in the degradation rate. The apparent activation energy of Arrhenius law for the sonodegradation of PCM is 10.3 kJ/mol, implying that the degradation reaction is indeed diffusion controlled [84]. As the frequency increases, the number of bubble oscillations per unit of time also increases, this would facilitate the mass transport of the pollutant molecules toward the cavitation bubbles [85], [86]. An increase in frequency also results in a higher population and smaller size cavitation bubbles, and consequently, more surface area for the adsorption and degradation of pollutant molecules. In addition, cavitation bubbles at higher frequencies are more stable [77] and live longer [87], providing more time for the mass transfer of pollutant molecules toward the cavitation bubble surface. These factors together could enhance the mass transfer of the PCM molecules from the solution bulk toward the cavitation bubbles. It should be noted that the DFUS at 780 kHz, belonging to the low ROS or SL zone, has better conformity to the trend in Fig. 12 (A) rather than that in Fig. 11 (A).

Despite the clear correlation between PCM degradation and HO• concentration, no correlation could be found between the overall SL intensity and the pollutant degradation (Fig. 12 B), implying that due to similar reasons, no considerable pyrolysis occurs in this zone as well, and the main mechanism for the degradation is by ROS action.

### 4.3 Correlation between degradation of paracetamol and SCL

**Fig. 13:**
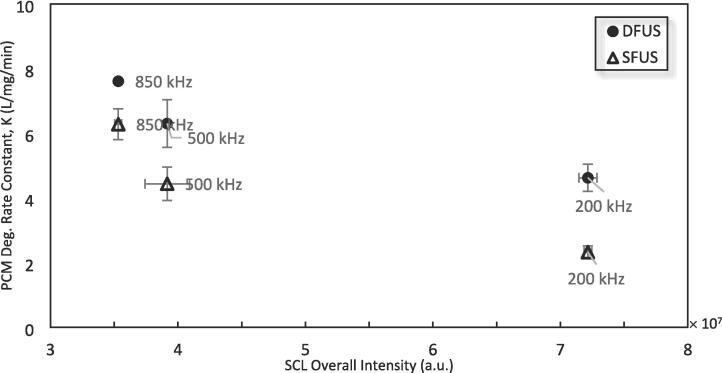
The relationship between the PCM degradation rate and the sonochemiluminescence (SCL) under SFUS and DFUS at 200, 500, and 850 kHz, as representatives of the low-, intermediate- and high-frequency zones.

Fig. 13 shows the PCM degradation rate versus the overall SCL intensities under SFUS and DFUS at 200, 500, and 850 kHz, as representatives of the low-, intermediate- and high-frequency zones. It could be seen that the increase in the SCL results in a decrease in the PCM degradation rate, which is counter intuitive and contradicts the trend observed with ROS in section 5.2.

As discussed in section 5.2 there exists a low and high zone in which the data would be better correlated under, but due to the selected frequencies studied under SCL, it is not possible to accurately determine if there is a similar SCL threshold. What is consistent with the discussion in 5.2.2 is that higher frequency ultrasound, despite a lower SCL intensity, induced a higher PCM degradation rate. This further substantiates the need for a comprehensive investigation on a range of different frequencies if the objective is to understand the effect of frequency.

## 5 Conclusions

For both SFUS and DFUS, the pseudo 2nd order kinetic model showed better conformity to the degradation data, which implies the high sensitivity of the heterogeneous sonodegradation reaction to the PCM bulk concentration, under the studied conditions. Also, the comparison of the degradation rate constants with the ROS yields as well as the overall SL intensities revealed the strong dependency on the yield of HO•, implying that the reaction site is on or in the vicinity of the surface of the cavitation bubbles. Despite the minimal effect of DFUS on the yield of HO•, the results showed that applying DFUS led to a synergistic effect in the degradation of PCM for all the degrading frequencies (200 – 1000 kHz). Considering the high sensitivity of the sonodegradation reaction to the PCM bulk concentration, the synergistic effect observed under DFUS could be attributed to the enhancement in mass transfer by introducing the 20 kHz ultrasound to the acoustic field. The decrease in the PCM degradation at high yields of HO• (due to the competition of H_2_O_2_ with PCM), as well as the enhancement observed for frequencies > 500 kHz despite having a low yield of HO• denote the dominance of the mass transfer resistance in controlling the reaction rate. With all these in mind, the improvement observed for degradation under DFUS, despite having a similar or lower HO• yield than SFUS, leads to the conclusion that the act of the 20 kHz ultrasound is reducing the mass transfer resistance for the heterogenous sonodegradation reaction, by facilitating the transport of pollutant molecules towards the cavitation bubbles. The results in this paper also demonstrates the importance of comprehensive characterisation of cavitation activity via different methods i.e., dosimetry, SL, and SCL to better elucidate the mechanism behind an application such as degradation of pollutants under SFUS and DFUS, and comparing between different frequencies.

## CRediT authorship contribution statement

**Mehrdad Zare:** Conceptualization, Methodology, Investigation, Writing – original draft, Writing – review & editing, Visualization. **Pello Alfonso-Muniozguren:** Conceptualization, Methodology, Investigation, Writing – review & editing. **Madeleine J. Bussemaker:** Writing – review & editing. **Patrick Sears:** Methodology, Resources, Writing – review & editing, Supervision. **Efraím A. Serna-Galvis:** Conceptualization, Writing – review & editing, Visualization. **Ricardo A. Torres-Palma:** Writing – review & editing, Supervision, Funding acquisition. **Judy Lee:** Conceptualization, Methodology, Resources, Writing – review & editing, Visualization, Supervision, Project administration, Funding acquisition.

## Declaration of Competing Interest

The authors declare that they have no known competing financial interests or personal relationships that could have appeared to influence the work reported in this paper.

## Data Availability

Data will be made available on request.

## References

[1] Gogoi A., Mazumder P., Tyagi V.K., Tushara Chaminda G.G., An A.K., Kumar M. (2018). Occurrence and fate of emerging contaminants in water environment: a review. Groundw. Sustain. Dev..

[2] Naddeo V., Belgiorno V., Ricco D., Kassinos D. (2009). Degradation of diclofenac during sonolysis, ozonation and their simultaneous application. Ultrason. Sonochem..

[3] Aristizabal-Ciro C., Botero-Coy A.M., López F.J., Peñuela G.A. (2017). Monitoring pharmaceuticals and personal care products in reservoir water used for drinking water supply. Environ. Sci. Pollut. Res..

[4] C.H. Bell, et al., Emerging contaminants handbook. 2019, Boca Raton, FL: CRC Press. 469.

[5] Emmanouil C., Bekyrou M., Psomopoulos C., Kungolos A. (2019). An insight into ingredients of toxicological interest in personal care products and a small-scale sampling survey of the Greek market: Delineating a potential contamination source for water resources. Water (Switzerland).

[6] Rubio-Clemente A., Torres-Palma R.A., Peñuela G.A. (2014). Removal of polycyclic aromatic hydrocarbons in aqueous environment by chemical treatments: a review. Sci. Total Environ..

[7] Tran N., Drogui P., Zaviska F., Brar S.K. (2013). Sonochemical degradation of the persistent pharmaceutical carbamazepine. J. Environ. Manage..

[8] Yap H.C., Pang Y.L., Lim S., Abdullah A.Z., Ong H.C., Wu C.-H. (2019). A comprehensive review on state-of-the-art photo-, sono-, and sonophotocatalytic treatments to degrade emerging contaminants. Int. J. Environ. Sci. Technol..

[9] A. Jelić, M. Petrović, D. Barcelo, Pharmaceuticals in drinking water. Emerging organic contaminants and human health, 2012: p. 47-70.

[10] Zhang Y., Geißen S.-U., Gal C. (2008). Carbamazepine and diclofenac: removal in wastewater treatment plants and occurrence in water bodies. Chemosphere.

[11] Daughton C.G., Ternes T.A. (1999). Pharmaceuticals and personal care products in the environment: agents of subtle change?. Environ. Health Perspect..

[12] Santos J.L., Aparicio I., Alonso E. (2007). Occurrence and risk assessment of pharmaceutically active compounds in wastewater treatment plants. A case study: Seville city (Spain). Environ. Int..

[13] Cleuvers M. (2004). Mixture toxicity of the anti-inflammatory drugs diclofenac, ibuprofen, naproxen, and acetylsalicylic acid. Ecotoxicol. Environ. Saf..

[14] Han S., Choi K., Kim J., Ji K., Kim S., Ahn B., Yun J., Choi K., Khim J.S., Zhang X., Giesy J.P. (2010). Endocrine disruption and consequences of chronic exposure to ibuprofen in Japanese medaka (Oryzias latipes) and freshwater cladocerans Daphnia magna and Moina macrocopa. Aquat. Toxicol..

[15] Gonzalez-Rey M., Bebianno M.J. (2012). Does non-steroidal anti-inflammatory (NSAID) ibuprofen induce antioxidant stress and endocrine disruption in mussel Mytilus galloprovincialis?. Environ. Toxicol. Pharmacol..

[16] Almeida Â., Calisto V., Esteves V.I., Schneider R.J., Soares A.M.V.M., Figueira E., Freitas R. (2014). Presence of the pharmaceutical drug carbamazepine in coastal systems: Effects on bivalves. Aquat. Toxicol..

[17] Jarvis A.L., Bernot M.J., Bernot R.J. (2014). The effects of the psychiatric drug carbamazepine on freshwater invertebrate communities and ecosystem dynamics. Sci. Total Environ..

[18] Schmidt W., O’Rourke K., Hernan R., Quinn B. (2011). Effects of the pharmaceuticals gemfibrozil and diclofenac on the marine mussel (Mytilus spp.) and their comparison with standardized toxicity tests. Mar. Pollut. Bull..

[19] Guiloski I.C., Ribas J.L.C., Pereira L.d.S., Neves A.P.P., Silva de Assis H.C. (2015). Effects of trophic exposure to dexamethasone and diclofenac in freshwater fish. Ecotoxicol. Environ. Saf..

[20] Rozman D., Hrkal Z., Váňa M., Vymazal J., Boukalová Z. (2017). Occurrence of pharmaceuticals in wastewater and their interaction with shallow aquifers: A case study of Horní Beřkovice, Czech Republic. Water.

[21] Homem V., Santos L. (2011). Degradation and removal methods of antibiotics from aqueous matrices – A review. J. Environ. Manage..

[22] Ikehata K., Jodeiri Naghashkar N., Gamal El-Din M. (2006). Degradation of aqueous pharmaceuticals by ozonation and advanced oxidation processes: a review. Ozone Sci. Eng..

[23] Wang J., Wang Z., Vieira C.L.Z., Wolfson J.M., Pingtian G., Huang S. (2019). Review on the treatment of organic pollutants in water by ultrasonic technology. Ultrason. Sonochem..

[24] J. Lee, Importance of sonication and solution conditions on the acoustic cavitation activity #5, in Handbook of Ultrasonics and Sonochemistry. 2016, Springer Singapore. p. 137-175.

[25] Brenner M.P., Hilgenfeldt S., Lohse D. (2002). Single-bubble sonoluminescence. Rev. Mod. Phys..

[26] Torres R.A., Pétrier C., Combet E., Carrier M., Pulgarin C. (2008). Ultrasonic cavitation applied to the treatment of bisphenol A. Effect of sonochemical parameters and analysis of BPA by-products. Ultrason. Sonochem..

[27] T.Y. Wu, et al., Theory and Fundamentals of Ultrasound, in Advances in Ultrasound Technology for Environmental Remediation, T.Y. Wu, et al., Editors. 2013, Springer Netherlands: Dordrecht. p. 5-12.

[28] Crum L.A. (1980). Measurements of the growth of air bubbles by rectified diffusion. J. Acoust. Soc. Am..

[29] Xu H., Eddingsaas N.C., Suslick K.S. (2009). Spatial separation of cavitating bubble populations: The nanodroplet injection model. J. Am. Chem. Soc..

[30] Rayaroth M.P., Aravind U.K., Aravindakumar C.T. (2016). Degradation of pharmaceuticals by ultrasound-based advanced oxidation process. Environ. Chem. Lett..

[31] Güyer G.T., Ince N.H. (2011). Degradation of diclofenac in water by homogeneous and heterogeneous sonolysis. Ultrason. Sonochem..

[32] Sivakumar M., Pandit A.B. (2001). Ultrasound enhanced degradation of Rhodamine B: optimization with power density. Ultrason. Sonochem..

[33] Ziylan A., Koltypin Y., Gedanken A., Ince N.H. (2013). More on sonolytic and sonocatalytic decomposition of Diclofenac using zero-valent iron. Ultrason. Sonochem..

[34] Ziylan-Yavas A., Ince N.H. (2018). Single, simultaneous and sequential applications of ultrasonic frequencies for the elimination of ibuprofen in water. Ultrason. Sonochem..

[35] Liu P. (2021). Sonochemical processes for the degradation of antibiotics in aqueous solutions: a review. Ultrason. Sonochem..

[36] Matafonova G., Batoev V. (2020). Dual-frequency ultrasound: strengths and shortcomings to water treatment and disinfection. Water Res..

[37] Zhang Y., Zhang Y., Li S. (2017). Combination and simultaneous resonances of gas bubbles oscillating in liquids under dual-frequency acoustic excitation. Ultrason. Sonochem..

[38] Wang S., Huang B., Wang Y., Liao L.i. (2006). Comparison of enhancement of pentachlorophenol sonolysis at 20 kHz by dual-frequency sonication. Ultrason. Sonochem..

[39] Barati A.H., Mokhtari-Dizaji M. (2010). Ultrasound dose fractionation in sonodynamic therapy. Ultrasound Med. Biol..

[40] Feng R., Zhao Y., Zhu C., Mason T.J. (2002). Enhancement of ultrasonic cavitation yield by multi-frequency sonication. Ultrason. Sonochem..

[41] Lee M., Oh J. (2011). Synergistic effect of hydrogen peroxide production and sonochemiluminescence under dual frequency ultrasound irradiation. Ultrason. Sonochem..

[42] Ninomiya K., Noda K., Ogino C., Kuroda S.-I., Shimizu N. (2014). Enhanced OH radical generation by dual-frequency ultrasound with TiO2 nanoparticles: Its application to targeted sonodynamic therapy. Ultrason. Sonochem..

[43] Yasuda K., Torii T., Yasui K., Iida Y., Tuziuti T., Nakamura M., Asakura Y. (2007). Enhancement of sonochemical reaction of terephthalate ion by superposition of ultrasonic fields of various frequencies. Ultrason. Sonochem..

[44] Zhao Y., Zhu C., Feng R., Xu J., Wang Y. (2002). Fluorescence enhancement of the aqueous solution of terephthalate ion after bi-frequency sonication. Ultrason. Sonochem..

[45] Zhu C., Feng R., Zhao Y. (2000). Sonochemical effect of a bifrequency irradiation. Chin. Sci. Bull..

[46] Brotchie A., Ashokkumar M., Grieser F. (2008). Sonochemistry and sonoluminescence under simultaneous high- and low-frequency irradiation. J. Phys. Chem. C.

[47] Gogate P.R., Sivakumar M., Pandit A.B. (2004). Destruction of Rhodamine B using novel sonochemical reactor with capacity of 7.5 l. Sep. Purif. Technol..

[48] Tiong T.J., Liew D.K.L., Gondipon R.C., Wong R.W., Loo Y.L., Lok M.S.T., Manickam S. (2017). Identification of active sonochemical zones in a triple frequency ultrasonic reactor via physical and chemical characterization techniques. Ultrason. Sonochem..

[49] Ebrahiminia A., Mokhtari-Dizaji M., Toliyat T. (2016). Dual frequency cavitation event sensor with iodide dosimeter. Ultrason. Sonochem..

[50] Suo D., Govind B., Zhang S., Jing Y. (2018). Numerical investigation of the inertial cavitation threshold under multi-frequency ultrasound. Ultrason. Sonochem..

[51] Ciuti P., Dezhkunov N.V., Francescutto A., Kulak A.I., Iernetti G. (2000). Cavitation activity stimulation by low frequency field pulses. Ultrason. Sonochem..

[52] Manickam S., Zainal Abidin N.B., Parthasarathy S., Alzorqi I., Ng E.H., Tiong T.J., Gomes R.L., Ali A. (2014). Role of H2O2 in the fluctuating patterns of COD (chemical oxygen demand) during the treatment of palm oil mill effluent (POME) using pilot scale triple frequency ultrasound cavitation reactor. Ultrason. Sonochem..

[53] Zhao L., Ma J., Zhai X. (2009). Synergetic effect of ultrasound with dual fields for the degradation of nitrobenzene in aqueous solution. Environ. Sci. Tech..

[54] Thanh Nguyen T., Asakura Y., Koda S., Yasuda K. (2017). Dependence of cavitation, chemical effect, and mechanical effect thresholds on ultrasonic frequency. Ultrason. Sonochem..

[55] Wu S., Zhang L., Chen J. (2012). Paracetamol in the environment and its degradation by microorganisms. Appl. Microbiol. Biotechnol..

[56] Paroj?i? J., Karljikovi?-Raji? K., Duri? Z., Jovanovi? M., Ibri? S. (2003). Development of the second-order derivative UV spectrophotometric method for direct determination of paracetamol in urine intended for biopharmaceutical characterisation of drug products. Biopharm. Drug Dispos..

[57] Hart E.J., Henglein A. (1985). Free radical and free atom reactions in the sonolysis of aqueous iodide and formate solutions. J. Phys. Chem..

[58] Alegria A.E., Lion Y., Kondo T., Riesz P. (1989). Sonolysis of aqueous surfactant solutions. Probing the interfacial region of cavitation bubbles by spin trapping. J. Phys. Chem..

[59] Awtrey A.D., Connick R.E. (1951). The absorption spectra of I2, I3-, I-, IO3-, S4O6= and S2O3=. Heat of the reaction I3-= I2+ I. J. Am. Chem. Soc..

[60] Okitsu K., Nanzai B., Kawasaki K., Takenaka N., Bandow H. (2009). Sonochemical decomposition of organic acids in aqueous solution: Understanding of molecular behavior during cavitation by the analysis of a heterogeneous reaction kinetics model. Ultrason. Sonochem..

[61] Calvisi M.L., Lindau O., Blake J.R., Szeri A.J. (2007). Shape stability and violent collapse of microbubbles in acoustic traveling waves. Phys. Fluids.

[62] Cairós C., Mettin R. (2017). Simultaneous high-speed recording of sonoluminescence and bubble dynamics in multibubble fields. Phys. Rev. Lett..

[63] Crum L.A., Reynolds G.T. (1985). Sonoluminescence produced by “stable” cavitation. J. Acoust. Soc. Am..

[64] Hatanaka S.-I., Mitome H., Yasui K., Hayashi S. (2002). Single-bubble sonochemiluminescence in aqueous luminol solutions. J. Am. Chem. Soc..

[65] Beckett M.A., Hua I. (2001). Impact of ultrasonic frequency on aqueous sonoluminescence and sonochemistry. Chem. A Eur. J..

[66] Koda S., Kimura T., Kondo T., Mitome H. (2003). A standard method to calibrate sonochemical efficiency of an individual reaction system. Ultrason. Sonochem..

[67] Okitsu K., Ashokkumar M., Grieser F. (2005). Sonochemical synthesis of gold nanoparticles: effects of ultrasound frequency. J. Phys. Chem. B.

[68] Yang L., Sostaric J.Z., Rathman J.F., Weavers L.K. (2008). Effect of ultrasound frequency on pulsed sonolytic degradation of octylbenzene sulfonic acid. J. Phys. Chem. B.

[69] Cravotto G., Cintas P. (2012). Harnessing mechanochemical effects with ultrasound-induced reactions. Chem. Sci..

[70] Lu X. (2021). A review on additives-assisted ultrasound for organic pollutants degradation. J. Hazard. Mater..

[71] J.A. Hangasky, et al., 5.12 - Glycosidic Bond Oxidation: The Structure, Function, and Mechanism of Polysaccharide Monooxygenases, in Comprehensive Natural Products III, H.-W. Liu and T.P. Begley, Editors. 2020, Elsevier: Oxford. p. 298-331.

[72] Villamena F.A., Villamena F.A. (2017). Reactive Species Detection in Biology.

[73] Wardman P. (1989). Reduction potentials of one-electron couples involving free radicals in aqueous solution. J. Phys. Chem. Ref. Data.

[74] Kanthale P., Ashokkumar M., Grieser F. (2008). Sonoluminescence, sonochemistry (H2O2 yield) and bubble dynamics: Frequency and power effects. Ultrason. Sonochem..

[75] Wood R.J., Lee J., Bussemaker M.J. (2019). Disparities between sonoluminescence, sonochemiluminescence and dosimetry with frequency variation under flow. Ultrason. Sonochem..

[76] Brotchie A., Grieser F., Ashokkumar M. (2009). Effect of power and frequency on bubble-size distributions in acoustic cavitation. Phys. Rev. Lett..

[77] Gaitan D.F., Crum L.A., Church C.C., Roy R.A. (1992). Sonoluminescence and bubble dynamics for a single, stable, cavitation bubble. J. Acoust. Soc. Am..

[78] T.E. Daubert, Physical and thermodynamic properties of pure chemicals: data compilation, in Design Institute for Physical Property Data (DIPPR). 1989, AIChE.

[79] Isariebel Q.-P., Carine J.-L., Ulises-Javier J.-H., Anne-Marie W., Henri D. (2009). Sonolysis of levodopa and paracetamol in aqueous solutions. Ultrason. Sonochem..

[80] Petrier C., Lamy M.-F., Francony A., Benahcene A., David B., Renaudin V., Gondrexon N. (1994). Sonochemical degradation of phenol in dilute aqueous solutions: comparison of the reaction rates at 20 and 487 kHz. J. Phys. Chem..

[81] Price G.J., Ashokkumar M., Grieser F. (2004). Sonoluminescence quenching of organic compounds in aqueous solution: frequency effects and implications for sonochemistry. J. Am. Chem. Soc..

[82] Wood R.J. (2020). Flow effects on phenol degradation and sonoluminescence at different ultrasonic frequencies. Ultrason. Sonochem..

[83] Guo W., Shi Y., Wang H., Yang H., Zhang G. (2010). Sonochemical decomposition of levofloxacin in aqueous solution. Water Environ. Res.

[84] Im J.-K., Boateng L.K., Flora J.R.V., Her N., Zoh K.-D., Son A., Yoon Y. (2014). Enhanced ultrasonic degradation of acetaminophen and naproxen in the presence of powdered activated carbon and biochar adsorbents. Sep. Purif. Technol..

[85] Campbell T.Y., Vecitis C.D., Mader B.T., Hoffmann M.R. (2009). Perfluorinated surfactant chain-length effects on sonochemical kinetics. J. Phys. Chem. A.

[86] Fyrillas M.M., Szeri A.J. (1996). Surfactant dynamics and rectified diffusion of microbubbles. J. Fluid Mech..

[87] Mestas J.-L., Lenz P., Cathignol D. (2003). Long-lasting stable cavitation. J. Acoust. Soc. Am..

