# Supplementary material for: A fundamental study on the degradation of paracetamol under single- and dual-frequency ultrasound
Source: Ultrason Sonochem. 2023 Feb 6;94:106320. doi: 10.1016/j.ultsonch.2023.106320 (PMC9925978; doi:10.1016/j.ultsonch.2023.106320)
Supplement: Supplementary Data 1 [file mmc1.docx]

**Supplementary data for:
A Fundamental study on the Degradation of Paracetamol under Single- and Dual-Frequency Ultrasound**

Mehrdad Zare^a^, Pello Alfonso-Muniozguren^a^, Madeleine J. Bussemaker^a^, Patrick Sears^a^, Efraím A. Serna-Galvis ^b,c^ , Ricardo A. Torres-Palma^b^, Judy Lee^a^

^a^ School of Chemistry and Chemical Engineering

University of Surrey, Guildford, GU2 7XH, United Kingdom

^b^ Grupo de Investigación en Remediación Ambiental y Biocatálisis (GIRAB), Instituto de Química, Facultad de Ciencias Exactas y Naturales, Universidad de Antioquia UdeA, Calle 70 # 52-21, Medellín, Colombia

^c^ Grupo de Catalizadores y Adsorbentes (CATALAD), Instituto de Química, Facultad de Ciencias Exactas y Naturales, Universidad de Antioquia UdeA, Calle 70 # 52-21, Medellín, Colombia

# Confirmation of the linearity of UV-Visible spectrophotometry and liquid chromatography (LC)/mass spectrometry results

To confirm the reliability of the UV-Visible spectrophotometer (UV-Vis.) and the liquid chromatography-mass spectrometry (LCMS), the linearity of the methods was investigated using standard solutions with concentrations ranging from 0.01 to 100 mg/L. Fig-S. 1 shows the results of the investigation. The results showed that the minimum limit of detection for both methods is 0.5 mg/L. Though the UV-Vis. shows more robustness and measures up to the concentration of 50 mg/L, which is ten times the maximum limit of detection for the LCMS method.

(A)

(B)

Fig-S. 1 – The results of the linearity test, for A) the UV-Vis., and B) the LCMS method. The linear fits and the corresponding equations and R^2^ are presented for the linear section of the curve.

# PCM Degradation: UV-Vis Results

The change in the initial concentration of PCM, as well as degradation intermediates (measured by UV-Vis.) as a function of the sonication time under SFUS and DFUS, is shown in Fig-S. 2 for selected frequencies. The 20 kHz horn alone did not cause any significant degradation of paracetamol. Similarly, the degradation rates for 22 kHz, 98 kHz, and 2 MHz were found to be zero under both SFUS and DFUS (data for 22 and 98 kHz not shown).

(A)

(B)

Fig-S. 2 – The change in normalised concentration of paracetamol solution with the initial concentration of 5 mg/L for 60 min, as a function of sonication time under A) SFUS, and B) DFUS. The concentration was measured using a UV-Vis. for all the frequencies, accounting for the change in the concentration of paracetamol and the degradation intermediates within the solution.

Pseudo 1^st^- and 2^nd^-order reaction kinetics models in terms of PCM bulk concentration were fitted to the degradation data. Table-S. 1 shows the coefficient of determination (R^2^) is high for both models. However, the sum of squared errors (SSE) for the pseudo 2^nd^ order model is at least one order of magnitude smaller for each frequency, suggesting that the model is more appropriate for interpreting the PCM degradation under the studied condition.

Table-S. 1– The Sum of Squared Errors (SSE) and the coefficient of determination (R^2^) calculated for pseudo-1^st^-order and pseudo-2^nd^-order reaction rate models, for degradation of paracetamol under SFUS and DFUS.

| Frequency (kHz) | SFUS | | | | DFUS | | | |
| --- | --- | --- | --- | --- | --- | --- | --- | --- |
|  | 1^st^ Order | | 2^nd^ Order | | 1^st^ Order | | 2^nd^ Order | |
|  | R^2^ | SSE × 10^4^ | R^2^ | SSE × 10^5^ | R^2^ | SSE × 10^4^ | R^2^ | SSE × 10^5^ |
| 200 | 0.986 | 2.20 | 0.970 | 0.49 | 0.993 | 3.19 | 0.985 | 0.93 |
| 300 | 0.997 | 0.94 | 0.995 | 0.25 | 0.999 | 0.50 | 0.997 | 0.23 |
| 400 | 0.987 | 5.53 | 0.960 | 2.15 | 0.996 | 2.27 | 0.990 | 0.90 |
| 500 | 0.977 | 7.22 | 0.925 | 3.26 | 0.992 | 8.20 | 0.979 | 2.81 |
| 780 | 0.996 | 2.76 | 0.986 | 1.46 | 0.987 | 10.90 | 0.976 | 3.68 |
| 850 | 0.995 | 4.35 | 0.992 | 0.84 | 0.997 | 3.13 | 0.991 | 1.44 |
| 1000 | 0.999 | 0.24 | 0.996 | 0.07 | 0.990 | 1.80 | 0.986 | 0.45 |

# PCM Degradation: LCMS Results

To confirm the accuracy of the spectrophotometry results as well as to account for the probable interference of intermediates, the sonicated solution of paracetamol was also analysed using a liquid chromatography/mass spectrometry (LCMS). An LCMS comprising Waters Acquity^TM^ Ultra-Performance Liquid Chromatograph (UPLC) equipped with a Kinetex® PS-C18 100 Å column (100 mm × 2.1 mm, 2.6 µm), followed by a Waters Zspray^TM^ single quadrupole mass spectrometer SQD2 (MS) was employed in positive electrospray ionisation (ESI+) mode.

According to the LCMS results, the change in the initial concentration of paracetamol as well as the intermediates as a function of sonication time under a single (SFUS) and dual (DFUS) frequency ultrasound is shown in Fig-S. 3.

Fig-S. 3 – The change in normalised concentration of paracetamol as a function of sonication time, measured by the LCMS under SFUS and DFUS at 500 kHz

The results for the application of pseudo-1^st^- and 2^nd^-order reaction kinetics models for the degradation data are shown in Fig-S. 3 are presented in Table-S. 2. The comparison of the coefficient of determination (R^2^) and the sum of squared errors (SSE) for the two models revealed that like for the UV-Vis. results, the pseudo-2^nd^-order model better described the LCMS results.

Table-S. 2 - The Sum of Squared Errors (SSE) and the coefficient of determination (R^2^) calculated for pseudo 1^st^ order and pseudo 2^nd^ order reaction rate, applied to the LCMS results for degradation of paracetamol under SFUS and DFUS at 500 kHz.

| Frequency (kHz) | SFUS | | | | DFUS | | | |
| --- | --- | --- | --- | --- | --- | --- | --- | --- |
|  | 1^st^ Order | | 2^nd^ Order | | 1^st^ Order | | 2^nd^ Order | |
|  | R^2^ | SSE | R^2^ | SSE | R^2^ | SSE | R^2^ | SSE |
| 500  (LCMS) | 9.98E-01 | 1.52E-03 | 9.98E-01 | 2.43E-05 | 9.99E-01 | 6.22E-04 | 9.98E-01 | 4.08E-05 |

# Evaluation of H_2_O_2_ yield

The yield of H_2_O_2_ (Fig-S. 4) was measured independently [1] and was compared to the H_2_O_2_ yield calculated by subtracting the HO• yield from the total ROS yield. For this purpose, 400 mL of pure MQ water was sonicated under DFUS at 500 kHz and 2.5 mL samples of freshly sonicated water were added to 1.25 mL of solution one (0.4 M KI, 0.1 M NaOH, 0.2 Mm (NH_4_)_6_Mo_7_O_24_.4H_2_O) and solution two (0.1 M KHC_8_H_4_O_4_).

The concentration of I_3_^-^, which is proportional to the concentration of H_2_O_2_ was determined by measuring the absorption at the wavelength of 350 nm using a UV-Vis. and based on the Beer-Lambert Law with a molar absorptivity (ε) of 26,000 L⋅mol^−1^⋅cm^−1^ and path length of 1 cm [2].

(A)

(B)

(C)

Fig-S. 4 - The concentration of I^-3^ yielded by H_2_O_2_, measured based on the method described by Alegria et al. [3] using UV-Vis. at the wavelength of 350 nm under DFUS with the power combination of 20 W and 30% at A) 200 kHz, B) 500 kHz, and C) 850 kHz.

The comparison of the H_2_O_2_ yield from this method with the calculated one (by subtracting the HO• yield from the yield of total ROS) mentioned in the article (Table-S. 3), shows that the results of both methods are acceptably close, showing the same trend.

Table-S. 3 – The comparison of the H_2_O_2_ yield measures ex-situ using the method described by Alegria et al. [3] versus the yield calculated by subtraction of HO• yield from the yield of total ROS, under DFUS with the power combination of 20 W and 30% at the mentioned frequencies.

|  | **H_2_O_2_ Yield** | | |
| --- | --- | --- | --- |
|  | **200 kHz** | **500 kHz** | **850 kHz** |
| Ex-situ measurement | 1.050 ± 0.059 | 0.779 ± 0.036 | 0.694 ± 0.011 |
| In-situ measurement ([Total ROS] - [HO•]) | 0.763 ± 0.023 | 0.623 ± 0.105 | 0.498 ± 0.089 |

# Synergistic index

The Synergistic Index (SI) for the yield of HO•, total ROS, and H_2_O_2_ is calculated based on Equation 2-1 of the article and plotted as a function of the frequency of the plate transducer in Fig-S. 5. For some frequencies the SI is less than or equal to one, meaning that applying the DFUS did not improve the yield of either of the ROSs. Also, no specific trend can be found in the graphs.

(A)

(B)

(C)

Fig-S. 5 – The synergistic index of A) HO• Yield, B) Total ROS Yield, and C) Total HO• Yield, calculated based on the Equation 2-1 of the article, and plotted as a function of frequency of the plate transducer.

# The Effect of the Ultrasonic Horn Body on the SL/SCL

In this section, the effect of the body of the ultrasonic horn, acting as a reflector in the acoustic field, is investigated on the overall intensity and the spatial distribution of SL and SCL.

Fig-S. 6 shows the overall intensities of SL and SCL at the studied frequencies under SFUS, having the ultrasonic horn off within the solution. A comparison of the data to the SFUS data shown in Fig. 5 of the article shows that the presence of the horn does not change the magnitude or the trend of the data points.

Fig-S. 6 – The overall SL/SCL intensities recorded for SFUS having the off ultrasonic horn inside the solution as a reflector.

Also, a comparison between Fig-S. 7 and Fig. 6 of the article confirms the same conclusion about the spatial distribution of SL and SCL. So, it could be concluded that the body of the horn acting as a wave reflector does not account for the effects observed under DFUS.

| **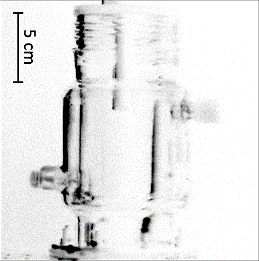**  The ultrasonic horn tip  The ultrasonic plate transducer | | | | | | | |
| --- | --- | --- | --- | --- | --- | --- | --- |
| **A) SL: SFUS + Horn (Off)** | | | |  |  |  |  |
| **22 kHz** | **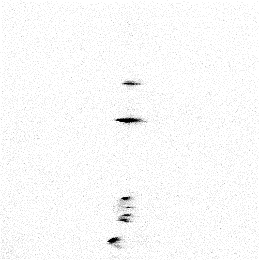** | **98 kHz** | **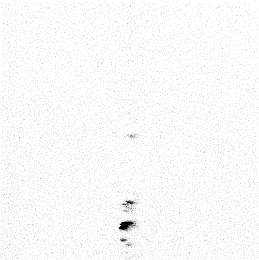** | **200 kHz** | **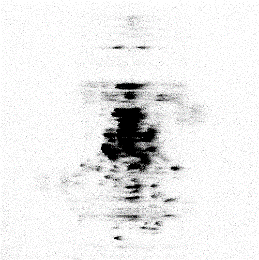** | **300 kHz** | **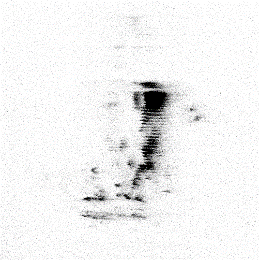** |
| **400 kHz** | **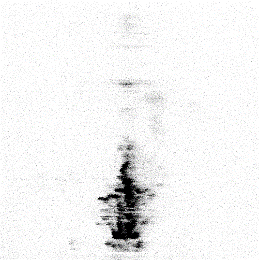** | **500 kHz** | **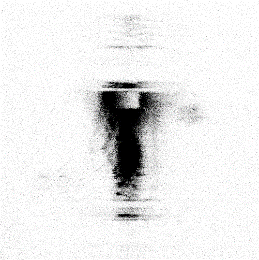** | **850 kHz** | **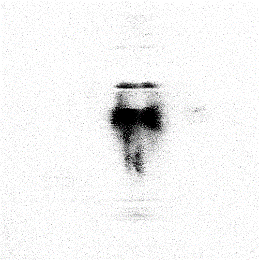** | **780 kHz** | **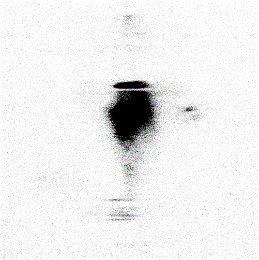** |
| **1000 kHz** | **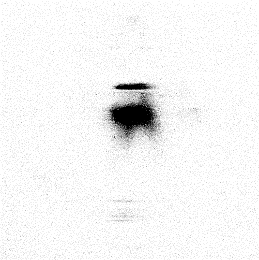** | **2000 kHz** | **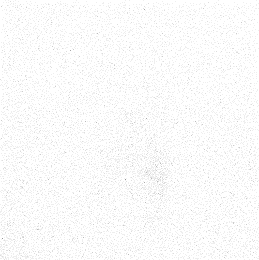** |  |  |  |  |
| **B) SCL: SFUS + Horn (Off)** | | | |  |  |  |  |
| **200 kHz** | **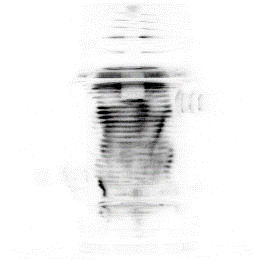** | **500 kHz** | **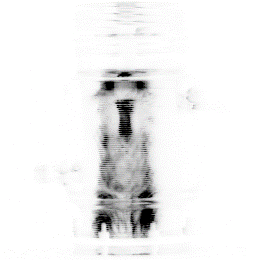** | **850 kHz** | **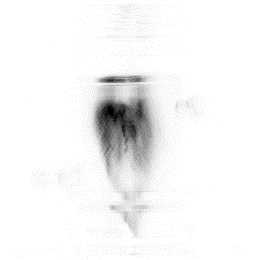** |  |  |

Fig-S. 7 – The spatial distribution of A) SL, and B) SCL, under SFUS having the horn off within the solution.

Fig-S. 8 shows the qualitative comparison of SL and SCL intensity and spatial distribution for SFUS and DFUS at various studied frequencies.

| **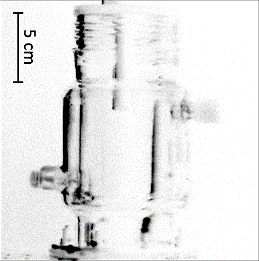**  The ultrasonic horn tip  The ultrasonic plate transducer | | | | | | | | | | |
| --- | --- | --- | --- | --- | --- | --- | --- | --- | --- | --- |
|  | **SFUS** | | **DFUS** | |  | |  | **SFUS** | | **DFUS** |
| **22 kHz** | **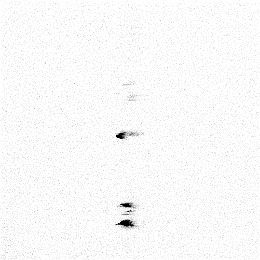** | | **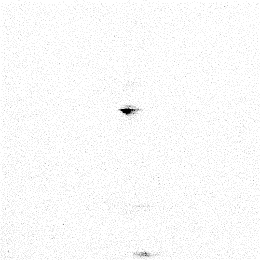** | |  | | **98 kHz** | **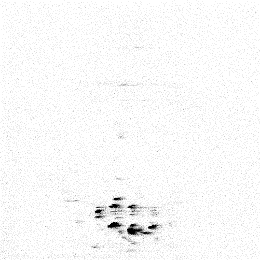** | | **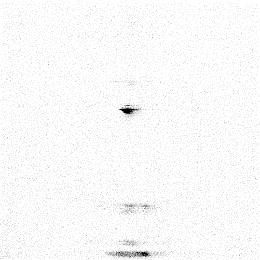** |
| **300 kHz** | **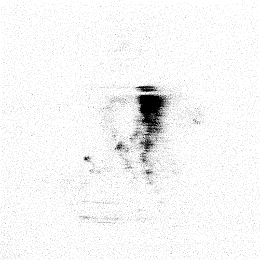** | | **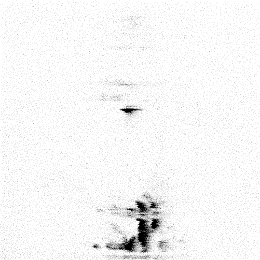** | |  | | **400 kHz** | **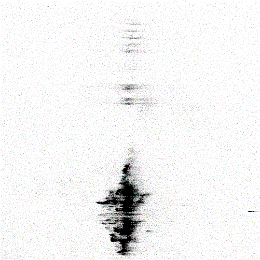** | | **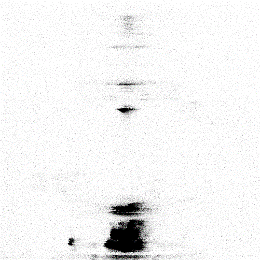** |
| **780 kHz** | **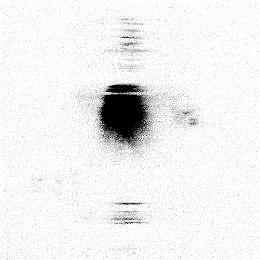** | | **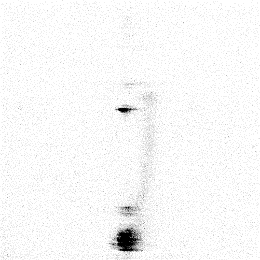** | |  | | **1000 kHz** | **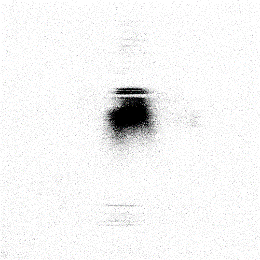** | | **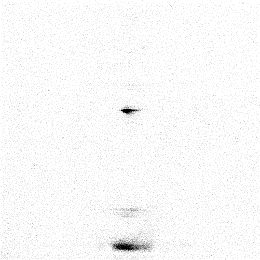**  The emission of SL/SCL due to the collapse of cavitation bubbles. |
|  | | **2000 kHz** | | **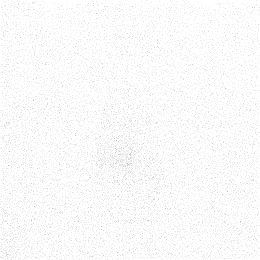** | | **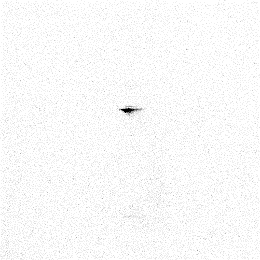** | | |  | |

Fig-S. 8 – Spatial distribution of Sonoluminescence (SL) emission imaged for SFUS and DFUS at various studied frequencies. For better visibility the images are inverted, and the black spots represent the SL/SCL. The picture of the empty reactor is presented as a guide/scale.

# Probability Density analysis for SL/SCL Intensities

In Fig-S. 9, the normal distribution function (NFD) for the SFUS and DFUS at 200 kHz, from the plateau section in Fig. 9 of the article is presented. Applying DFUS increases the average bubble radius and pushes the curve to the right. Also, it makes the curve wider suggesting a wider bubble size distribution (larger standard deviation). This suggests that introducing the 20 kHz to the acoustic field of frequencies of the plateau in Fig. 9 promotes the growth of the bubbles, resulting in larger and more diverse bubble diameters.

Fig-S. 9 – The probability density function for the sonoluminescence intensities measured by each camera pixel under SFUS and DFUS at 200 kHz, belonging to the plateau section of Fig. 9.

In Fig-S. 10, the NDF for the frequencies of the linear section in Fig. 9 of the article is presented. Applying DFUS shifts the maximum of the curve (average bubble size) to the left and reduces the curve width (smaller standard deviation), suggesting a decrease in the average and distribution of cavitation bubble diameters. It can be concluded that for these frequencies, applying DFUS limits the growth of the bubbles, resulting in smaller and more uniform bubbles. It is noteworthy that despite the 500 kHz belonging to the plateau section of Fig. 9, it shows a similar behaviour under DFUS, meaning that DFUS makes the active cavitation bubbles smaller and more uniform (Fig-S. 10 C).

Also, in Fig-S. 10 (F) the NDF for the SL recorded for the 20 kHz horn is presented. It seems that the SFUS at 2 MHz does not generate considerable SL and based on the similarity between the curves of the DFUS at 2MHz and the 20 kHz horn, the majority of the SL intensities recorded for the DFUS at 2MHz can be attributed to the ultrasonic horn.

In Fig-S. 11, the results of the same analysis for the SCL intensities recorded for 200, 500, and 850 kHz are presented. The results do not show a distinct meaningful behaviour for the SCL intensities, such as that discussed for the SL intensities

(A)

(B)

(C)

(D)

(E)

(F)

Fig-S. 10 – The probability density function for the SL intensities measured by each camera pixel under SFUS and DFUS of A) 22 kHz, B) 98 kHz, C) 500 kHz, D) 780 kHz, E) 1 MHz, and F) 2 MHz and 20 kHz horn, from the linear section of Fig. 9 of the article.

(A)

(B)

(C)

Fig-S. 11 – The probability density function for the SCL intensities measured by each camera pixel under SFUS and DFUS of A) 200 kHz, B) 500 kHz, and C) 850 kHz.

# Correlation between degradation of paracetamol and HO•, H_2_O_2_ and SL intensity

The degradation of paracetamol versus the yield of HO• as well as the overall SL intensity is presented in Fig-S. 12.

(A)

(B)

Fig-S. 12 – The degradation of paracetamol versus A) HO· Yield, and B) the overall SL intensity

The HO• yield shows a better correlation with the degradation. However, the plot for the total ROS yield is presented as well in Fig-S. 13, for the Low and High ROS and SL zones.

(A)

(B)

Fig-S. 13 – The degradation of paracetamol versus the total ROS yield for A) Low SL or ROS zone, and B) High SL or ROS zone (The ROS and SL threshold considered to define the low and high zones are 1 μM/min and 1×10^7^ a.u., respectively)

# References

1. Allen, A.O., et al., *Oak Ridge National Laboratory Publication.* ORNL, 1949. **130**.

2. Hart, E.J. and A. Henglein, *Free radical and free atom reactions in the sonolysis of aqueous iodide and formate solutions.* Journal of Physical Chemistry, 1985. **89**(20): p. 4342-4347.

3. Alegria, A.E., et al., *Sonolysis of aqueous surfactant solutions. Probing the interfacial region of cavitation bubbles by spin trapping.* Journal of Physical Chemistry, 1989. **93**(12): p. 4908-4913.
